# Candidate genes underlying hypomelanistic morphs in squamate reptiles

**DOI:** 10.1093/genetics/iyaf236

**Published:** 2025-11-03

**Authors:** Pierre Beaudier, Asier Ullate-Agote, Athanasia C Tzika

**Affiliations:** Laboratory of Artificial and Natural Evolution, Department of Genetics and Evolution, University of Geneva, Geneva 1205, Switzerland; Laboratory of Artificial and Natural Evolution, Department of Genetics and Evolution, University of Geneva, Geneva 1205, Switzerland; Laboratory of Artificial and Natural Evolution, Department of Genetics and Evolution, University of Geneva, Geneva 1205, Switzerland

**Keywords:** reptiles, skin, skin coloration, melanin, hypomelanism, snakes, lizards, pigmentation

## Abstract

Skin coloration is crucial for the survival of animals and ranges from spectacular colorful displays used to attract a mate to cryptic camouflage used to avoid predators. Among the 3 main types of chromatophores, melanophores are the most widespread in vertebrates and can set the skin tone by the amount of melanin they produce and store in dedicated vesicles, the melanosomes. Mutations associated with melanophore differentiation and maturation result in hypomelanistic and amelanistic phenotypes, both extensively studied in mammals but less so in snakes and lizards. Here, we characterize at the genomic, transcriptomic, and histological level, the Hypomelanistic corn snake morph and 3 hypomelanistic leopard gecko morphs. To minimize bias in studying leopard gecko color morphs, we first assembled a chromosome-level genome from a wild-type individual in terms of coloration. We propose that candidate mutations in 3 melanogenesis factors generate these phenotypes: (i) tyrosinase (*TYR*), an essential enzyme for melanin synthesis, (ii) NCKX5 (*SLC24A5*), an ion exchanger involved in melanosome maturation, and (iii) the P protein (*OCA2*), a transmembrane transporter for tyrosine. Our extended bulk RNA sequencing analyses show that additional pigmentation-related genes, affecting melanin production, melanosome motility, and melanophore migration, are dysregulated in the embryonic skin of the mutated animals. This observation highlights the likely associations among the corresponding pathways and is in line with our electron microscopy imaging results. Indeed, the subcellular structure of melanophores is uniquely altered at each of the 4 morphs and likely reflects a multigenic effect. These findings demonstrate that conserved pigmentation genes can produce species-specific effects, underscoring the modular nature of skin coloration in vertebrates. Our work establishes reptiles as comparative models for studying pigment cell biology and reveals evolutionary flexibility in the genetic regulation of melanogenesis.

## Graphical Abstract

**Graphical Abstract:**
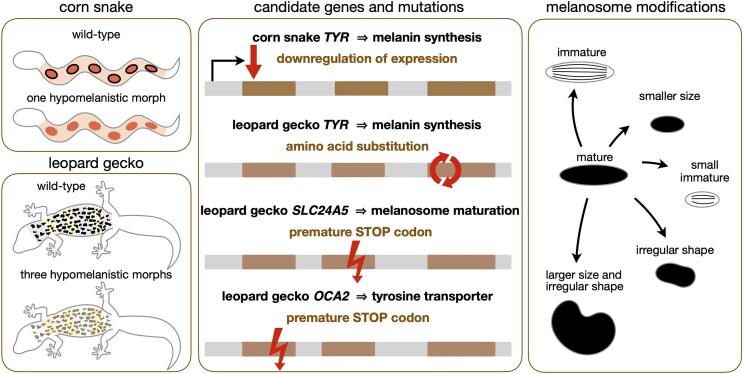

## Teaser

The study investigated how genetic changes affect skin coloration in reptiles, a topic less explored than in mammals. Researchers analyzed corn snakes and leopard geckos with reduced pigmentation, called hypomelanism. They used genome sequencing, gene expression analysis, and microscopy to examine pigment cells. The results showed that mutations in genes controlling melanin production and melanosome development cause lighter coloration. Each reptile morph displayed distinct changes in pigment cell structure. The study concludes that the same pigmentation genes can act differently across species. These findings establish reptiles as valuable models for studying skin color and its evolutionary diversity in vertebrates.

## Introduction

The coloration of skin appendages plays an important role in the individual's interactions with its environment through the colors and patterns displayed. These characteristics enable intra- and inter-species communication, such as recognition, sexual status, aposematism, camouflage, and mimicry (Hofreiter and Schoneberg 2010), and also fulfill physiological functions (Cuthill et al. 2017). Variations can impact the ability of an individual to survive and are therefore subject to selective pressure. Thus, coloration traits provide an excellent model to study adaptive evolution and the genetic and developmental mechanisms that determine it. In vertebrates, coloration is primarily produced by chromatophores. These cells originate from the neural crest and migrate in the skin during embryogenesis, where they differentiate and interact with each other and their environment to generate color patterns (Bagnara 1983). Melanophores and xanthophores carry black and yellow or red pigments, respectively, while iridophores produce arrays of guanine nanocrystals, which, through light interference, can produce a wide range of colors.

Among chromatophores, the mechanism of melanin production in melanophores is the most studied, as these are the only type of chromatophores in mammals—named melanocytes—and they are implicated in several human diseases (Yamaguchi and Hearing 2014; Snyman et al. 2024). The key reaction involves the tyrosinase enzyme (TYR), which first hydroxylates tyrosine into L-3,4-dihydroxyphenylalanine (L-DOPA) and subsequently oxidizes L-DOPA into dopaquinone (Korner and Pawelek 1982). This compound will then be used to synthesize melanin. The reaction takes place in melanosomes, lysosome-related organelles that go through 4 stages of maturation. Stage I premelanosomes are nonpigmented vacuoles of endosomal origin that contain the premelanosome protein (PMEL). This transmembrane glycoprotein is proteolytically processed in the melanosome into fragments that will form amyloids. The amyloids will in turn assemble into sheets, reorganizing the lumen and forming a functional amyloid matrix where melanin will be deposited in later stages (Graham et al. 2019). In stage II, intraluminal striations composed of PMEL start to form and are visible by electron microscopy. Melanin is synthesized and deposited upon the striations in Stage III. Finally, in stage IV, striations are not visible anymore and the mature melanosomes appear black because of melanin accumulation (D’Alba and Shawkey 2019; Le et al. 2021). Melanogenesis is tightly controlled by multiple genetic, hormonal, or environmental factors mainly by regulating: (i) tyrosinase availability (Bae-Harboe and Park 2012; Zolghadri et al. 2019), (ii) proper melanosome formation (Fujita et al. 2009), and (iii) vesicle trafficking (Ohbayashi and Fukuda 2020). Studies of melanogenesis in other vertebrate model species, such as the zebrafish, have found homologous elements performing similar cellular functions (Camp and Lardelli 2001; Lister et al. 2001; Beirl et al. 2014; Patterson and Parichy 2019). However, there have been very few studies of this process in reptiles, which remain largely understudied compared with other vertebrates. Research on color mutants with disrupted black/brown coloration confirmed the involvement of known melanogenesis actors such as the P protein (*OCA2* gene) and PMEL in the Amelanistic and Terrazzo corn snakes, respectively (Ullate-Agote et al. 2014; Saenko et al. 2015; Tzika et al. 2024), and the tyrosinase (*TYR* gene), the P protein (*OCA2* gene), the tyrosinase-related protein 1 (*TYRP1* gene), and the melanocortin 1 receptor (*MC1R* gene) in 4 ball python color morphs (Brown et al. 2022; Garcia-Elfring et al. 2025), or the tyrosinase in the Japanese rat snake (Iwanishi et al. 2018). Although the primary elements of melanogenesis are likely conserved, mutations of its components could impact reptilian coloration differently within the clade and compared with other vertebrates.

Here, we focus on hypomelanistic traits of the corn snake (*Pantherophis guttatus*) and the leopard gecko (*Eublepharis macularius*), 2 species of the Squamata order. The corn snake is a nonvenomous oviparous species from the southeastern USA with a wild-type pattern made up of dorsal and lateral red blotches on an orange background, while the ventral side features black and white checkers. The leopard gecko is a small nocturnal lizard native to the dry and desertic regions of Pakistan, Afghanistan, Iran, India, and Nepal; its wild-type pattern consists of irregular black spots on a yellow background dorsally and a uniform white ventral side (Agarwal et al. 2022). They are both popular pets, and several color and pattern morphs have emerged in private collections over time (Love and Love 2005; Tremper and Tremper 2019). The systematic genetic characterization of these morphs makes it possible to identify the key players responsible for the diversity of color patterns encountered in reptiles (Saenko et al. 2015; Ullate-Agote et al. 2020; Guo et al. 2021; Tzika et al. 2024; Ullate-Agote and Tzika 2024; Montandon et al. 2025). Leopard geckos are also model species for regeneration and temperature-dependent sex determination among others (reviewed in Agarwal et al. 2022).

**Fig. 1.**
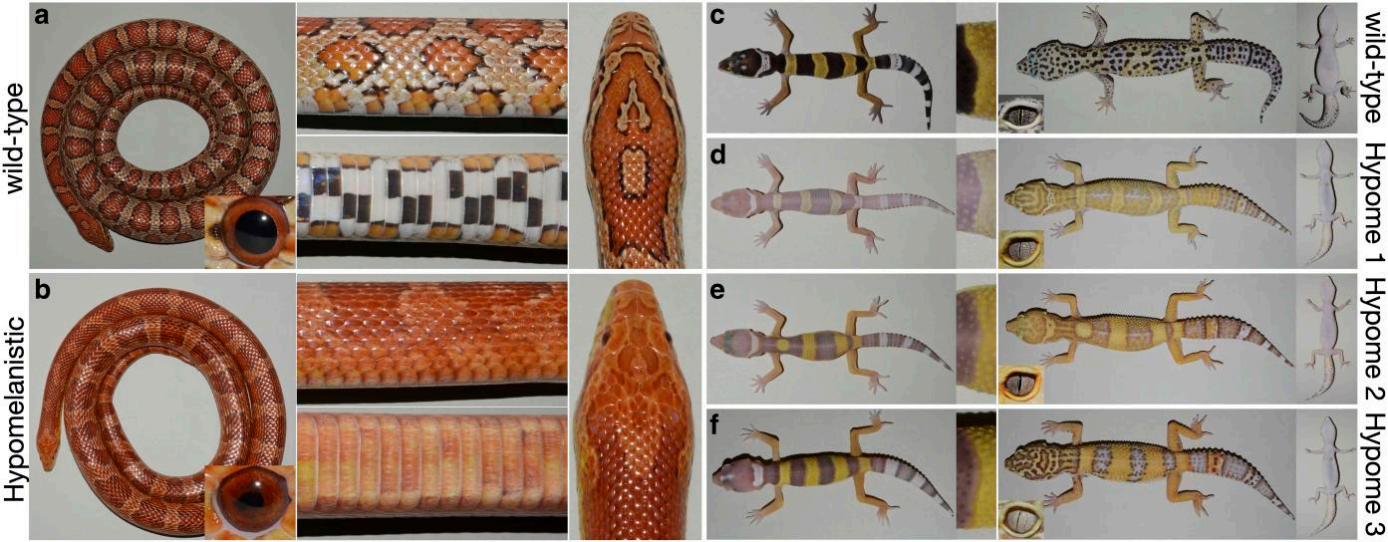
Hypomelanistic corn snake and leopard geckos. a) The corn snake wild-type pattern consists of red dorsal and lateral blotches on an orange background and features black and white ventral checkers. The round pupil is black, and the iris is red. b) In Hypomelanistic corn snakes, the black contour of the blotches is reduced, the black checkers are less evident, and the shape of the pupil appears irregular. c) Wild-type leopard geckos hatch with a banded pattern that changes to spots during the first year of their life. The elliptical pupil is black, and the iris is silver with black strikes. In Hypome 1 d), Hypome 2 e), and Hypome 3 f) leopard geckos, we primarily observe that the amount of melanin is reduced in the skin and the eyes.

We characterized at the genetic, transcriptomic, and histological level, the Hypomelanistic corn snake morph and 3 hypomelanistic leopard gecko morphs: (i) the Tremper Albino, (ii) the Rainwater Albino, and (iii) the Bell Albino (Fig. 1). For simplicity, the leopard gecko morphs are referred to as Hypome 1, Hypome 2, and Hypome 3, respectively, throughout the text. These traits appeared in captive stocks in the 1980s and late 1990s and were all identified to be mono-locus and recessive (Love and Love 2005; Tremper and Tremper 2019). Using mapping-by-sequencing, we identified the genomic interval harboring these mutations and investigated the expression profile of the candidate genes during embryonic development using bulk RNA sequencing. For the corn snake, we have produced extensive genomic resources (Ullate-Agote et al. 2014, 2017, 2020), with a chromosome-level assembly recently published (Hoge et al. 2024). For the leopard gecko, a high-quality RefSeq assembly (GCF_028583425.1) is available (Pinto et al. 2023), but the reference individual was homozygous for the Hypome 1 (Tremper Albino) allele, heterozygous for the incomplete dominant Lemon Frost allele, a mutation impacting iridophores (Guo et al. 2021), and possibly heterozygous for the Murphy patternless allele, a mutation that affects pattern formation. To avoid any bias in the investigation of leopard gecko color morphs, we present here a new chromosome-level assembly generated from a wild-type—in terms of coloration—individual using a combination of short and long sequencing reads and chromatin conformation information. Based on our findings, the Hypomelanistic corn snake phenotype is associated with the downregulation of *TYR*, while a missense mutation in the same gene might be responsible for the Hypome 1 leopard gecko phenotype. The Hypome 2 and Hypome 3 leopard gecko phenotypes are associated with disruptive mutations in the *SLC24A5* (Solute carrier family 24 member 5) and *OCA2* (Oculocutaneous albinism II) genes, respectively. Finally, our electron microscopy imaging revealed the impact of each mutation on the subcellular structure of the melanosomes. In conclusion, we link known melanin-related genes to 4 hypomelanistic morphs of 2 squamates—that is lizards and snakes—and examine how their disruption or dysregulation affects the production of melanin and alters their skin coloration.

## Material and methods

### Animals and ethics statement

Corn snakes and leopard geckos were housed and bred at the LANE animal facility running under the veterinary cantonal permit no. 1008. Sampling and imaging were performed under the experimentation permits GE2719/29163, GE24/33145, and GE366/36450 approved by the Geneva Canton ethical regulation authority.

### Leopard gecko genome assembly

Blood was collected from a 1-year-old adult female leopard gecko with wild-type coloration. DNA was extracted using the DNA Blood mini kit (Qiagen, 51104) for PacBio HiFi and Illumina Paired-end libraries, and with Arima Genomic Genome Assembly Hi-C kit for Illumina Hi-C libraries. For the assembly, we used the *pb_assembly_hifi* module from the SmrtLink v12.0 software suite (https://www.pacb.com/smrt-link/). To further improve the assembly, the Hi-C data were processed with the Arima genomics mapping pipeline (https://github.com/ArimaGenomics/mapping_pipeline). The resulting BAM files were then processed together using *YAHS* v1.1 (Zhou et al. 2023) for Hi-C scaffolding, generating the final genome assembly (NCBI Accession GCA_050574515.1). Gene-content quality control was carried out using *BUSCO* v5.4.4 (Manni et al. 2021) with the *vertebrata_odb10* dataset.

The assembly was annotated with LiftOn v1.0.5 (Chao et al. 2025), which combines DNA-DNA alignments from Liftoff (Shumate and Salzberg 2021) and protein-DNA alignments from miniprot (Li 2023) to map annotations between genome assemblies. We used the NCBI reference genome assembly and RefSeq annotation as input (GCF_028583425.1). The resulting annotation is composed of 23,389 genes and 34,804 transcripts (see *Data Availability*).

### Genomic DNA next-generation sequencing

Genomic DNA was extracted from the parents and the offspring of both corn snakes and leopard geckos using the DNeasy Blood and Tissue kit (Qiagen, 69504) following the manufacturer's instructions. We used blood, sheds, or muscle as starting material. The TruSeq DNA PCR-free libraries were sequenced using an Illumina HiSeqX instrument producing 150 bp paired-end reads. We obtained 171 to 200 million reads for the corn snake libraries and 148 to 258 million reads for the leopard gecko libraries. We checked the data quality and presence of adapters with FastQC (Andrews 2010). We performed quality filtering with sickle v1.33 (Joshi NA 2011), we retained between 158 and 185 million reads for the corn snake libraries, which corresponds to a 28×–32.7× average coverage for a 1.7 Gb genome, and between 144 and 262 million reads for the leopard gecko libraries, which corresponds to a 19.7×–35.8× average coverage for a 2.2 Gb genome.

### SNP calling and genomic interval mapping

The genomic libraries of each morph were assigned to their respective reference genomes (GCF_029531705.1 for corn snake, GCA_050574515.1 for leopard gecko) using *bwa* v0.7.17 (Li and Durbin 2009) with default parameters in *mem* mode. We used *SAMtools* v1.9 (Danecek et al. 2021) to: (i) convert the output SAM files into BAM, (ii) remove duplicates using the *fixmate* mode with the *-m* flag and the *markdup* mode with the *-r* flag, and (iii) sort out reads by their leftmost coordinates. Repetitive regions of the genome were identified using *RepeatMasker* v4.1.5 (Smit et al. 2013–2015) and variants from these regions were ignored. We identified genomic variants with *Platypus* v0.8.1 (Rimmer et al. 2014) and retrieved the genomic interval where the phenotype locus is located as previously described (Ullate-Agote et al. 2020). We predicted the impact of each co-segregating genomic variant found within the interval on genes and proteins using the *SnpEff* toolbox (Cingolani et al. 2012).

### Bulk RNA-Seq sampling and analysis

We first extracted genomic DNA from each embryo, and we genotyped them by verifying the presence of fixed SNPs in amplified and Sanger-sequenced fragments at the edge of the corresponding intervals. The primers for the amplification and sequencing are provided in Supplementary Table 1 in Supplementary File 2. Only the Hypome 2 samples were not genotyped. We then extracted total RNA from the dorsal skin of corn snake and leopard gecko samples using the Direct-zol RNA MiniPrep (Zymo Research, R2050). For the hypomelanistic and Hypome 3 samples the RNA integrity number (RIN) was ≥9, but it varied for the Hypome 1 and Hypome 2 samples. 100 bp paired-end TruSeq Stranded mRNA libraries were prepared for each sample and produced between 40.8 and 50 million reads for the corn snake libraries and between 19.2 and 30 million reads for the leopard gecko libraries.

The bulk RNA-Seq samples were aligned to the CU assembly (GCF_029531705.1.1) for the corn snake and to our assembly (GCA_050574515.1) for the leopard gecko with *STAR* (v2.7.10b) (Dobin et al. 2013) using default parameters for paired-end libraries. Gene expression quantification was performed using the *featureCounts* function implemented in the *Subread* package (v2.0.6) (Liao et al. 2013) counting uniquely mapped paired-end reads. Data normalization, transformation (considering variance stabilizing transformation), principal component analysis, and differential expression analyses with the Wald test and an FDR of 0.05 were performed with the *DESeq2* package (v1.42.0) (Love et al. 2014). For the Hypome 2 samples, which were produced from 2 breeding pairs, we included the batches in the design of the *DESeq2* experiment to account for the batch effect.

### Transmission electron microscopy

Samples were prepared as previously described (Ullate-Agote et al. 2020). In short, skin pieces of 1 mm^2^ were fixed in 2.5% glutaraldehyde solution (EMS), postfixed by a fresh mixture of osmium tetroxide 1% (EMS) with 1.5% potassium ferrocyanide (Sigma-Aldrich), dehydrated in acetone solutions (Sigma-Aldrich) and infiltrated in Epon (Sigma-Aldrich) at graded concentrations. Transveral ultrathin sections of 50 nm were cut on a Leica Ultracut (Leica Mikrosysteme) and poststained with 2% uranyl acetate (Sigma). Micrographs were taken with a transmission electron microscope (Philips CM100; Thermo Fisher Scientific).

## Results

### Coloration of a Hypomelanistic corn snake and three hypomelanistic leopard geckos

In contrast to the wild-type corn snakes, hypomelanistic individuals have a barely discernible black contour around their blotches, whereas the red coloration of the blotches and the orange coloration of the background are conserved (Fig. 1, a and b). At the ventral side, the black and white checkers are maintained in the hatchlings, but the black checkers have a red tint (Supplementary Fig. 1, a and b in Supplementary File 1). On the adults’ ventral side, we observe a fairly homogenous red pigmentation where faint traces of the black checkers are still visible, a phenomenon that also occurs in adult wild-type individuals (Supplementary Fig. 1c in Supplementary File 1). Although the intensity of the black in the pupil is similar to the wild-type, the distribution or number of melanophores is impacted giving it an irregular shape instead of circular.

In hypomelanistic leopard geckos, we observe variation in the intensity of their black coloration (Fig. 1, c to f, Supplementary Fig. 2 in Supplementary File 1), but the yellow pigmentation is not impacted by the respective mutations. In the eyes of the 3 morphs, the pupil maintains its black color and elliptical shape, but it becomes thinner. In the silver iris, we observe less strikes, which take a light brown color in Hypome 3. Note that in private collections there is great variation in the coloration intensity as well as the pattern of these morphs which is likely due to the diverse genetic background of the animals. Our own crosses and the ones performed by private breeders (Tremper and Tremper 2019) confirmed that these 3 morphs correspond to 3 different mutations that are mono-locus and recessive. Furthermore, a past study confirmed the presence of functional tyrosinase in the 3 mutants, although the amount probably varies (Gamble et al. 2006).

In the lineages of Hypomelanistic corn snakes and leopard geckos we maintain, there are no major pattern modifications compared to the respective wild-types. Thus, we assume that melanophores are present and their number is not drastically impacted. Indeed, coloration patterns take shape during embryogenesis as chromatophores differentiate. The chromatophores interact with each other to form the blotches of the corn snakes and the bands of the leopard gecko hatchlings. We have previously shown that in these squamate species, for the pattern to be established the 3 types of chromatophores need to be present in sufficient numbers (Tzika et al. 2024; Ullate-Agote and Tzika 2024) and capable of effective interactions (Montandon et al. 2025). In the hypomelanistic animals under study, we hypothesize that melanophores properly migrate and differentiate during embryogenesis and it is primarily melanogenesis that is affected.

### *TYR* is downregulated in hypomelanistic corn snakes

We first performed mapping by sequencing to retrieve the genomic locus harboring the causative variant for the Hypomelanistic corn snake phenotype, as previously described (Ullate-Agote et al. 2020). Libraries were produced from 1 homozygous and 1 heterozygous parent and 2 pools of homozygous and heterozygous offspring, respectively (Supplementary Table 2 in Supplementary File 2). The reads were aligned to the reference corn snake assembly (NCBI accession: GCF_029531705.1). For each library, we retained only the variants corresponding to the expected genotype, that is, homozygous variants in libraries from homozygous individuals and heterozygous variants in libraries from heterozygous snakes. We then computed the percentage of co-segregating variants transmitted from parents to offspring in sliding windows of 1 Mb. The genomic region carrying the causative mutation is expected to exhibit the highest percentage of co-segregation.

**Fig. 2.**
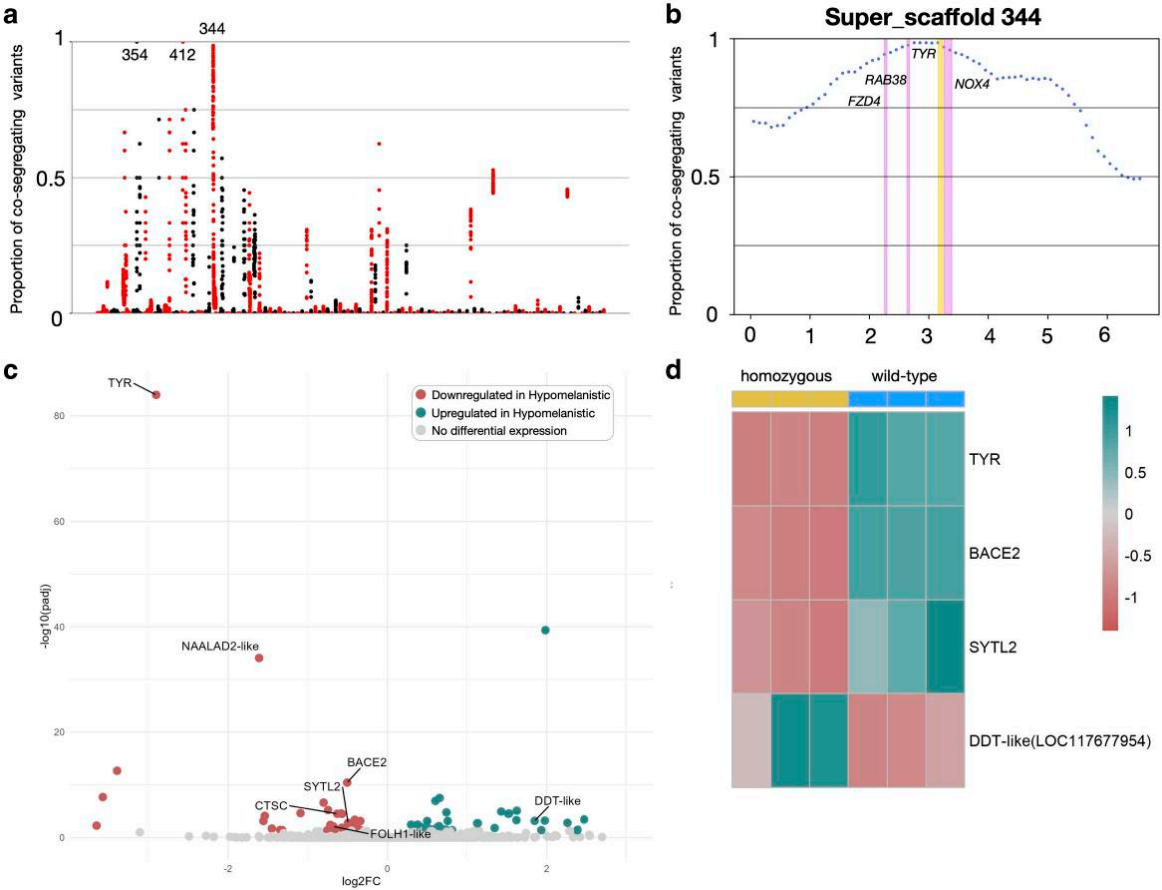
Genetic and transcriptomic analyses for the Hypomelanistic corn snake morph. a) Proportion (*y*-axis) of quality-filtered biallelic SNP/MNP co-segregating with the hypomelanistic locus in the 4 genomic libraries compared to informative quality-filtered parental variants. Proportions are calculated for scaffolds >1 Mb, with a 1-Mb sliding window and a step of 100 Kb. Scaffolds (alternatively colored black and red) are ordered from longest to shortest. We label Super-scaffolds 344, 354, and 412. b) Proportion of biallelic variants (SNP/MNP and indels) in 200-Kb intervals with a 50-Kb step co-segregating with the hypomelanistic locus in Super-scaffold 344. We highlight in yellow the position of *TYR* and in red the positions of *RAB38*, *FZD4,* and *NOX4*. c) Volcano plot depicting statistically significant gene expression changes between wild-type and homozygous hypomelanistic embryonic dorsal skin samples in terms of log2 fold-change (*x*-axis) and negative log10 of adjusted *P* value (*y*-axis). In cyan, the genes significantly upregulated in hypomelanistic and in red, the ones significantly downregulated (cutoff adjusted *P* value: 0.05). We label the differentially expressed genes situated in the reduced genomic interval and the ones associated with melanogenesis. d) Heatmap of genes that are associated with melanogenesis and differentially expressed in the hypomelanistic samples.

Peaks of high co-segregation can be seen on 3 scaffolds (Fig. 2a). We disregarded the peaks on Super-scaffolds 354 and 412 because they correspond to regions with a low number of co-segregating variants (only 3 and 9, respectively). The third peak with more than 85.6% co-segregation spans 2 Mb on Super-scaffold 344 (from 1.85 to 3.85 Mb) and 5,967 co-segregating variants are present (Fig. 2b). Among the 16 genes found within this interval, 6 genes present non-synonymous substitutions of one or multiple amino acids, but no high-impact variants resulting in severe protein disruptions, such as premature STOP codons and intron/donor acceptor site mutations, were detected (Supplementary Table 3 in Supplementary Files 2 and 3). According to InterProScan (Jones et al. 2014), only one missense mutation results in a protein domain modification: loss of the “*FUNFAM: NADPH oxidase 4 domain*” in *NOX4* (NADPH Oxidase 4). NOX4 generates reactive oxygen species that have been found to reduce melanin formation in mouse melanoma cells, possibly by inhibiting the synthesis of tyrosinase (Liu et al. 2012). Thus, a nonfunctional NOX4 would result in an increased amount of melanin and increased *TYR* expression, which is not the case in Hypomelanistic corn snakes. Three additional genes linked to melanogenesis are present in the interval: *TYR* and *RAB38* (RAB38 member of the RAS oncogene family), both well-known key genes in melanin production, and *FZD4* (Frizzled Class Receptor 4), a member of the Wnt/β-catenin signaling pathway, which is involved in melanocyte expansion and differentiation (Dunn et al. 2000).

In addition to the genomic characterization, we performed differential expression analyses by extracting total RNA from the dorsal skin of 3 homozygous hypomelanistic (*m^h^*/*m^h^*) and 3 wild-type (+/+) embryos at embryonic day 40, corresponding to the developmental stage 10 as defined for *Boaedon (Lamprophis) fuliginosus* (Boback et al. 2012) (Supplementary Table 4 in Supplementary File 2). At this stage, melanin is present in the skin. To minimize variance due to the diverse genetic background of the parents, all embryos were obtained from a single clutch of a cross between 2 heterozygous parents (*m^h^*/+*×m^h^*/+). According to the principal component analyses (PCA), the main differentiating factor of the samples at this stage is the sex of the embryos (PC1: 46%) as we have previously seen in this type of transcriptomic analyses during embryonic development (Tzika et al. 2024; Montandon et al. 2025) (Supplementary Fig. 3a in Supplementary File 1). Among the 81 genes differentially expressed between the wild-type and the hypomelanistic samples (Fig. 2c, Supplementary Table 5 in Supplementary File 2), 4 are located in the genomic interval: *TYR*, *CTSC* (Cathepsin C), *LOC117660188* (*NAALAD2*-like), and *LOC117660097* (*FOLH1*-like). Only *TYR* is expressed by chromatophore precursors according to our previously published single-cell transcriptomic dataset for the dorsal skin of an embryo at embryonic day 25 (Tzika et al. 2024) (Supplementary Fig. 3b in Supplementary File 1). The other genes may be expressed by chromatophores at other developmental stages. *FOLH1* encodes a transmembrane glycoprotein acting as a glutamate carboxypeptidase. It belongs to the family of NAALADases, which is notably involved in the regulation of the NAAG neurotransmitter, but no link has been found to pigmentation for this gene (Zink et al. 2020). *NAALAD2* encodes a protein with a similar function of NAAG regulation; mutations in this gene have been associated with the disruption of retinal pigment epithelial cells (Qian et al. 2025) but not with skin pigmentation. Hypomelanistic corn snakes do not present noticeable changes in their behavior due to poor eyesight compared to wild-types, although, if *LOC117660188* (*NAALAD2*-like) were differentially expressed in their eyes, fundus photography and histological analyses would be necessary to verify if their eye morphology is impacted. Cathepsin C is a peptidase involved in serine protease activation and it was found to be specifically expressed in melanocytes of the neck skin of the black-bone chicken (Wang et al. 2024). Its function in the melanogenesis pathway has not been established, but it could play a similar role to other cathepsins expressed in melanocytes by maintaining melanosome homeostasis (Rebelo et al. 2024) and assisting their degradation (Homma et al. 2018). In a human patient, deficiency in Cathepsin C resulted in the Papillon–Lefevre syndrome affecting skin pigmentation, but a concrete link with melanogenesis pathways is lacking (Ikeshima 2006). The other genes of interest in the interval, *NOX4*, *FZD4,* and *RAB38*, are not differentially expressed, with *NOX4* being very lowly expressed in wild-type and hypomelanistic samples (Supplementary Fig. 3c in Supplementary File 1). Based on our genomic and transcriptomic analyses, we conclude that most likely a regulatory mutation reducing the expression level of *TYR* in Hypomelanistic corn snakes is responsible for their modified skin phenotype. The disruption of NOX4 does not appear to be a plausible candidate mutation, as it would have caused hypermelanism and increased expression of *TYR*, neither of which occurs in Hypomelanistic corn snakes. *CTSC* is a less-likely candidate because it is not expressed by chromatophores at the developmental stage sampled, although we cannot exclude its expression in melanophores later in development.

Beyond *TYR* and the genes in the genomic interval, we investigated whether genes involved in melanogenesis and pigmentation are also differentially expressed. For this purpose, we compiled a list of 505 genes associated with gene ontology terms including the words “*tyrosinase,*” “*melanin,*” “*melanosome,*” “*melanocyte,*” and “*pigmentation*” in human (*Homo sapiens*), mouse (*Mus musculus*), zebrafish (*Danio rerio*), frog (*Xenopus tropicalis*), chicken (*Gallus gallus*), and anole lizard (*Anolis carolinensis*) using AmiGO 2 (Carbon et al. 2009) (Supplementary Table 6 in Supplementary File 2). From those, we observed (i) the downregulation of *BACE2* (Beta-secretase 2), an enzyme processing the PMEL into functional amyloids (Rochin et al. 2013), (ii) the downregulation of *SYTL2* (Synaptotagmin-like 2), required for peripheral melanosome distribution and elongated melanocyte shape (Kuroda and Fukuda 2004), and (iii) the upregulation of *DTT* (D-dopachrome decarboxylase—*LOC117677954*), an enzyme directly involved in melanin synthesis (Odh et al. 1993) (Fig. 2d, Supplementary Fig. 3d in Supplementary File 1). Obviously, such analyses reveal associations, that is, proteins jointly contributing to a shared function, but it does not necessarily mean that they are physically binding each other. The abnormal expression of a candidate gene can impact the expression of other pigmentation-related genes directly, if it functions as a transcription factor, or indirectly via a feedback loop. In Hypomelanistic corn snakes, the downregulation of *TYR* might activate a feedback loop that leads to the altered expression of *BACE2, SYTL2,* and *DTT.* This could occur either by direct interactions, as TYR, BACE2, and SYTL2 colocalize in the melanosome, or indirectly since DTT is primarily found in the cytoplasm. A change in the number of chromatophores could also explain the differential expression of pigmentation-related genes in the mutated phenotype. However, this parameter is difficult to assess in the nontransparent skin of corn snakes. The associations revealed here imply that not only the melanin synthesis is impacted in Hypomelanistic corn snakes but also potentially the subcellular structure of melanophores.

### Leopard gecko genome assembly from a wild-type individual

The current reference genome of the leopard gecko (NCBI accession: GCF_028583425.1) was assembled from an individual carrying several coloration-related mutations that can influence mapping-by-sequencing analyses. Indeed, relevant genomic intervals might be mutated, absent, or translocated thus preventing the proper identification of candidate genes. Therefore, we produced a new genome assembly of a 1-year-old adult female displaying wild-type coloration and, to our knowledge, carrying no pigmentation-related mutations. Three sequencing technologies were combined to produce the assembly: 1.6 million PacBio HiFi reads and 2.67 billion Illumina paired-end short reads were assembled in contigs, and a Hi-C library (3.7 billion Illumina reads) supported scaffolding (Supplementary Table 7 in Supplementary File 2). The final genome assembly is 2.23 Gbp long consisting of 313 scaffolds, 20 of them having chromosome length (NCBI Accession GCA_050574515.1). The quality metrics are similar to those of the reference assembly (Table 1). We used LiftOn (Chao et al. 2025) to transfer the NCBI annotation of the reference assembly to our own (see *Data Availability*). We performed the mapping-by-sequencing analyses for the Hypome 1, Hypome 2, and Hypome 3 phenotypes using our own genome.

**Table 1.** Quality metrics of leopard gecko genome assemblies.

	Scaffold N50	Scaffold L50	Sequencing depth	Complete BUSCO	Fragmented BUSCO	Missing BUSCO
Reference assembly	145 Mb	6	30.0×	98.0% [S:96.2%, D:1.8%]	0.8%	1.2%
New assembly	144 Mb	5	195.0×	97.7% [S:95.9%, D:1.8%]	0.9%	1.4%

Gene-content quality metrics were obtained using BUSCO (vertebrata_odb10 dataset) on the reference genome assembly of the leopard gecko (NCBI accession: GCF_028583425.1) and the newly produced genome assembly (NCBI Accession: GCA_050574515.1). S stands for single-copy genes and D for duplicated genes.

### A *TYR* missense mutation associated with the Hypome 1 phenotype

**Fig. 3.**
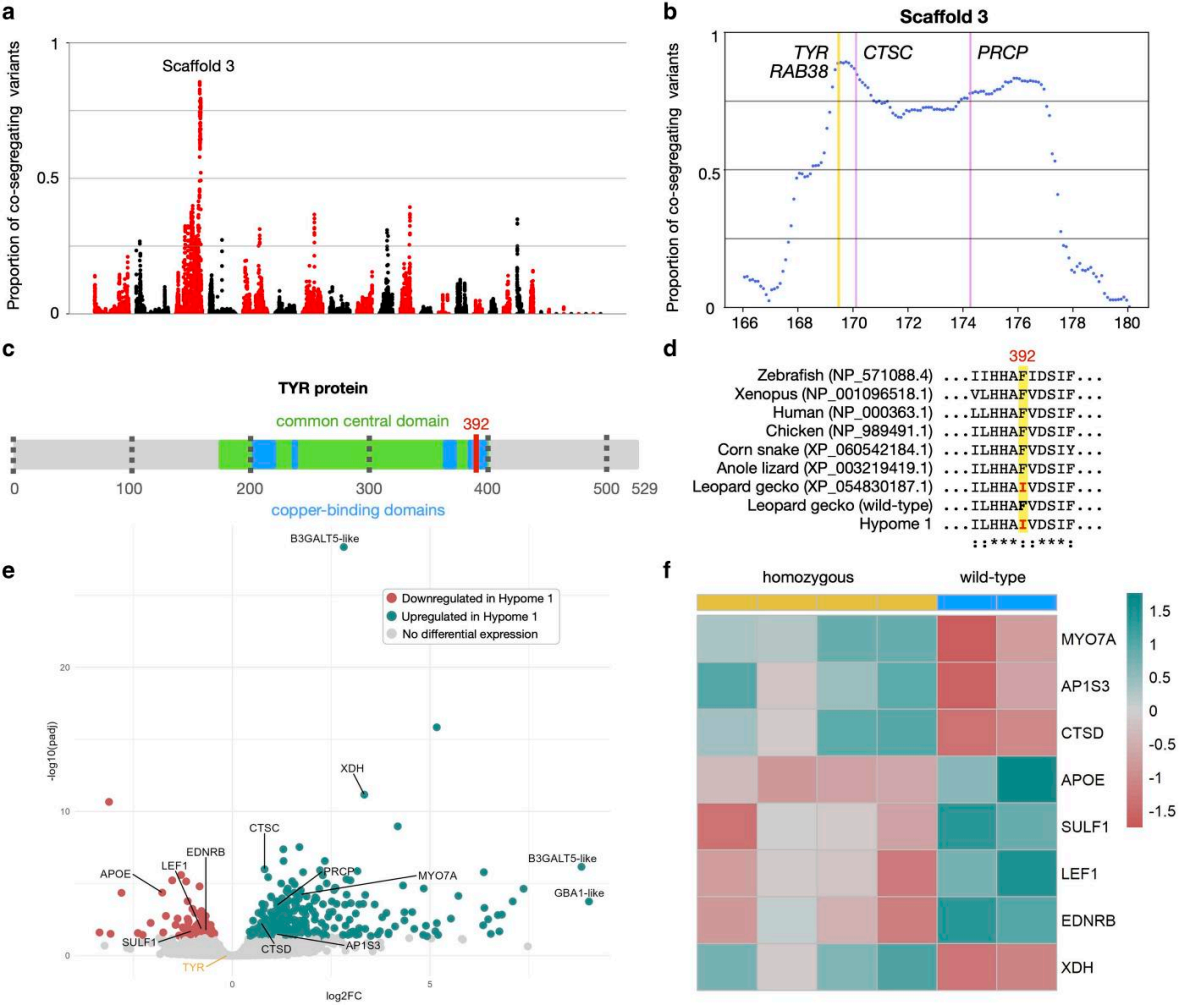
Genetic and transcriptomic analyses for the Hypome 1 leopard gecko morph. a) Proportion (*y*-axis) of quality-filtered biallelic SNP/MNP co-segregating with the Hypome 1 locus in the 4 genomic libraries compared to informative quality-filtered parental variants. Proportions are calculated for scaffolds >1 Mb, with a 1-Mb sliding window and a step of 100 Kb. Scaffolds (alternatively colored black and red) are ordered from longest to shortest. We label Scaffold 3. b) Proportion of biallelic variants (SNP/MNP and indels) in 200-Kb intervals with a 50-Kb step co-segregating with the Hypome 1 locus on Scaffold 3. We highlight in yellow the position of *TYR*. c) Schematic representation of the leopard gecko TYR protein. The position of the 2 mutations is indicated with a red line. d) Amino acid alignment of the TYR protein surrounding the missense mutation. We use “*” to indicate conserved sites and “:” for conservative sites where an amino acid is replaced by another of similar properties. e) Volcano plot depicting statistically significant gene expression changes between wild-type and homozygous Hypome 1 embryonic dorsal skin samples in terms of log2 fold-change (*x*-axis) and negative log10 of adjusted *P* value (*y*-axis). In cyan, the genes significantly upregulated in Hypome 1 and in red, the ones significantly downregulated (cutoff adjusted *P* value: 0.05). We label the differentially expressed genes associated with melanogenesis and the genes with the greatest fold change and *P* value. f) Heatmap of genes that are associated with melanogenesis and differentially expressed in the Hypome 1 samples.

Mapping-by-sequencing for the Hypome 1 phenotype was performed as described for the Hypomelanistic corn snake (Supplementary Table 2 in Supplementary File 2). Only one region with a percentage of co-segregation variants greater than 69.1% was found on Scaffold 3 (Fig. 3a). It ranges from 169.1 to 177 Mb, with the highest levels of co-segregation (89.3%) between 169.1 and 170.3 Mb (Fig. 3b). This is also the range with the greatest number of co-segregating variants (Supplementary Fig. 4a in Supplementary File 1) and it likely carries the causative mutation for Hypome 1. In addition, we performed extended mapping analyses that included the 4 family libraries, as well as sequencing data of the RefSeq individual that was also a Hypome 1 homozygous. We retrieve the same short interval on Chromosome 3 (Supplementary Fig. 4b in Supplementary File 1).

The large 7.9 Mb region contains 51 genes, and the short interval of 1.2 Mb only 9 (Supplementary Table 8 in Supplementary File 2). Although none of these genes carries a high-impact mutation, we find amino acid substitutions in 7 genes (Supplementary File 3), none of which impact the predicted protein domains according to InterProScan. Among the 9 genes in the short interval, we find *RAB38* and *TYR*, both of which are also present in the genomic interval of the Hypomelanistic corn snake morph. We identified one amino acid modification at position 392 of the Hypome 1 leopard gecko TYR, which does not affect the protein domain prediction, as previously stated. We cannot exclude though an impact on the enzymatic function as it falls on the “common central domain of tyrosinase” domain predicted by PFAM (Mistry et al. 2021) and the overlapping “tyrosinase copper-binding domain signature” domain predicted by PRINTS (Attwood et al. 2012) (Fig. 3c). A multispecies alignment of the leopard gecko tyrosinase protein with that of other vertebrates revealed that this is a highly conserved position among vertebrates. It is only mutated in Hypome 1 individuals and the RefSeq individual that was identified as Hypome 1 (Fig. 3d, Supplementary Fig. 4c in Supplementary File 1).

We performed bulk RNA-Seq on dorsal skin samples from 2 wild-type (+/+), 4 homozygous (*m^h1^*/*m^h1^*), and 3 heterozygous (*m^h1^*/+) embryos at developmental stage 41 (Wise et al. 2009) (Supplementary Table 4 in Supplementary File 2). This stage corresponds roughly to embryonic day 40 post oviposition, when melanin production has initiated on the embryonic skin. Note that leopard gecko females lay 6 to 8 clutches of only 2 eggs throughout the breeding season, unlike corn snakes that lay a single clutch of 15 to 20 eggs. We chose to collect embryos from a single pair of heterozygous (*m^h1^*/+) parents to avoid differences due to the genetic background of the parents, so the sampling occurred over several seasons. The PCA clustering clearly separates the homozygous samples from the heterozygous and wild-type ones (PC1 variance: 53%—Supplementary Fig. 5a in Supplementary File 1). The eggs were incubated at a temperature that mostly produces females. As the embryos cannot be sexed morphologically, we cannot assess the impact of sex on the differential expression analyses. In the wild-type versus homozygous comparison (Fig. 3e), 410 genes are differentially expressed, 2 of which are in the large genomic interval (Supplementary Table 9 in Supplementary File 2): *PRCP* (Prolylcarboxypeptidase), and *CTSC* (Cathepsin C). The 3 most upregulated genes, in terms of fold change (*B3GALT5-like* and *GBA1-like*) and *P*-value (*LOC129326753- B3GALT5-like*) have not been associated with skin pigmentation so far. In the heterozygous versus homozygous comparison (Supplementary Fig. 5b in Supplementary File 1, Supplementary Table 10 in Supplementary File 2), 1,069 genes are differentially expressed, including again *PRCP* and *CTSC* from the larger genomic interval.

*PRCP* is involved in the α-MSH (α-melanocyte stimulating hormone) degradation (Diano 2011), a neuropeptide stimulating the production and release of melanin in the skin. The over-expression of *PRCP* in Hypome 1 leopard geckos could lead to lower levels of α-MSH and in turn lower amount of melanin produced by melanocytes in the skin. Note that we also observe *PRCP* over-expression in heterozygous samples compared to the wild-type but with a lower log2 fold change (heterozygous: 0.57, homozygous: 1.13). The absence of a hypomelanistic phenotype in heterozygous geckos could be explained by a threshold value of *PRCP* expression above which melanogenesis would be impacted and which would be exceeded for the homozygous mutation (Supplementary Fig. 5c in Supplementary File 2). We previously generated single-cell transcriptomic data from the dorsal skin of a leopard gecko embryo at an embryonic stage when melanin starts to be produced (Ullate-Agote and Tzika 2024). At this specific developmental stage, *PRCP* is mostly expressed by endothelial cells and not chromatophores (Supplementary Fig. 5d in Supplementary File 2).

As stated earlier for the Hypomelanistic corn snakes, *CTSC* is likely involved in melanin production, but it is unknown how. It was found specifically expressed by melanocytes of black-feathered chicken and downregulated in the skin of white-feathered chicken. In leopard geckos, we observe expression in wild-type chromatophores (Supplementary Fig. 5d in Supplementary File 1), but its upregulation in Hypome 1 leopard geckos would rather result in hypermelanism. Although we cannot exclude the involvement of *PRCP* and *CTSC* in the Hypome 1 phenotype, we hypothesize that the skin phenotype of the animals is altered primarily by reduced function of TYR. Indeed, *TYR* is situated on the interval with the greatest proportion of co-segregating SNPs and is primarily expressed by melanophores (Supplementary Fig. 5d in Supplementary File 1).

We also assessed the expression of the 505 selected melanogenesis-associated genes (Fig. 3f, Supplementary Fig. 5, e and f in Supplementary File 1), with *MYO7A* (Myosin VIIA), *AP1S3* (Adaptor-related protein complex 1 subunit sigma 3), and *CTSD* (Cathepsin D) being overexpressed in the homozygous Hypome 1 and *APOE* (Apolipoprotein E), *SULF1* (Sulfatase 1), *LEF1* (Lymphoid enhancer-binding factor 1), and *EDNRB* (Endothelin receptor type B) being downregulated. *MYO7A* is expressed in mouse retinal pigment cells and mutations cause the Usher syndrome 1B resulting, among others, in melanosome motility defects, but only in retinal pigment cells and not in skin melanocytes (Williams and Lopes 2011). AP1S3 is a subunit of the clathrin adaptor protein AP-1, which creates, along with KIF13A, endosomal subdomains in melanocytes necessary for cargo delivery of enzymes, such as *TYRP1*, to mature melanosomes (Delevoye et al. 2009). The knockdown of Cathepsin D in zebrafish larvae has been linked to skin hyperpigmentation with the dispersion of the melanophores over the yolk. The process leading to this phenotype has not been precisely identified and it could not be studied in adult fish as the larvae die at 10 d post-fertilization (Follo et al. 2011). APOE is a regulator of the amyloid fibrils’ formation by PMEL via its binding to exosomes and intraluminal vesicles. Also, it likely plays a role in protecting pigment cells from the inherent toxicity of amyloidogenesis (van Niel et al. 2015). Expression of *SULF1* is necessary for the chromatophore migration from the neural crest and their subsequent patterning in zebrafish (Meyers et al. 2013). *LEF1* encodes an enhancer involved in the regulation of *TYR* expression, both by increasing *MITF* expression and by enhancing the promoter activity of *TYR* (Wang et al. 2015). As *MITF* is involved in the differentiation of chromatophores from the neural crest cells, *LEF1* might also play a significant role in this process (Dorsky et al. 2000). EDNRB is a key factor in the migration of melanoblasts from the neural crest (Lee et al. 2003), which is regulated by *MITF* (Sato-Jin et al. 2008). We thus observe a dysregulation of factors that affect both the migration and the differentiation of melanophores, as well as the production of melanin.

Among the upregulated genes in homozygous Hypome 1 leopard geckos (Fig. 3f), we also find *XDH,* which is involved in pteridine pigment synthesis in xanthophores (Parichy et al. 2000). Its overexpression hints at a possible increase in the production of yellow pigments and/or the number of xanthophores. This change can be a response to the decrease of melanin production. Note though that leopard geckos in the pet trade often carry multiple mutations affecting their coloration, and the increased expression of *XDH* could be linked to another mutation present in the lineage studied.

### *SLC24A5* is disrupted in Hypome 2 leopard geckos

**Fig. 4.**
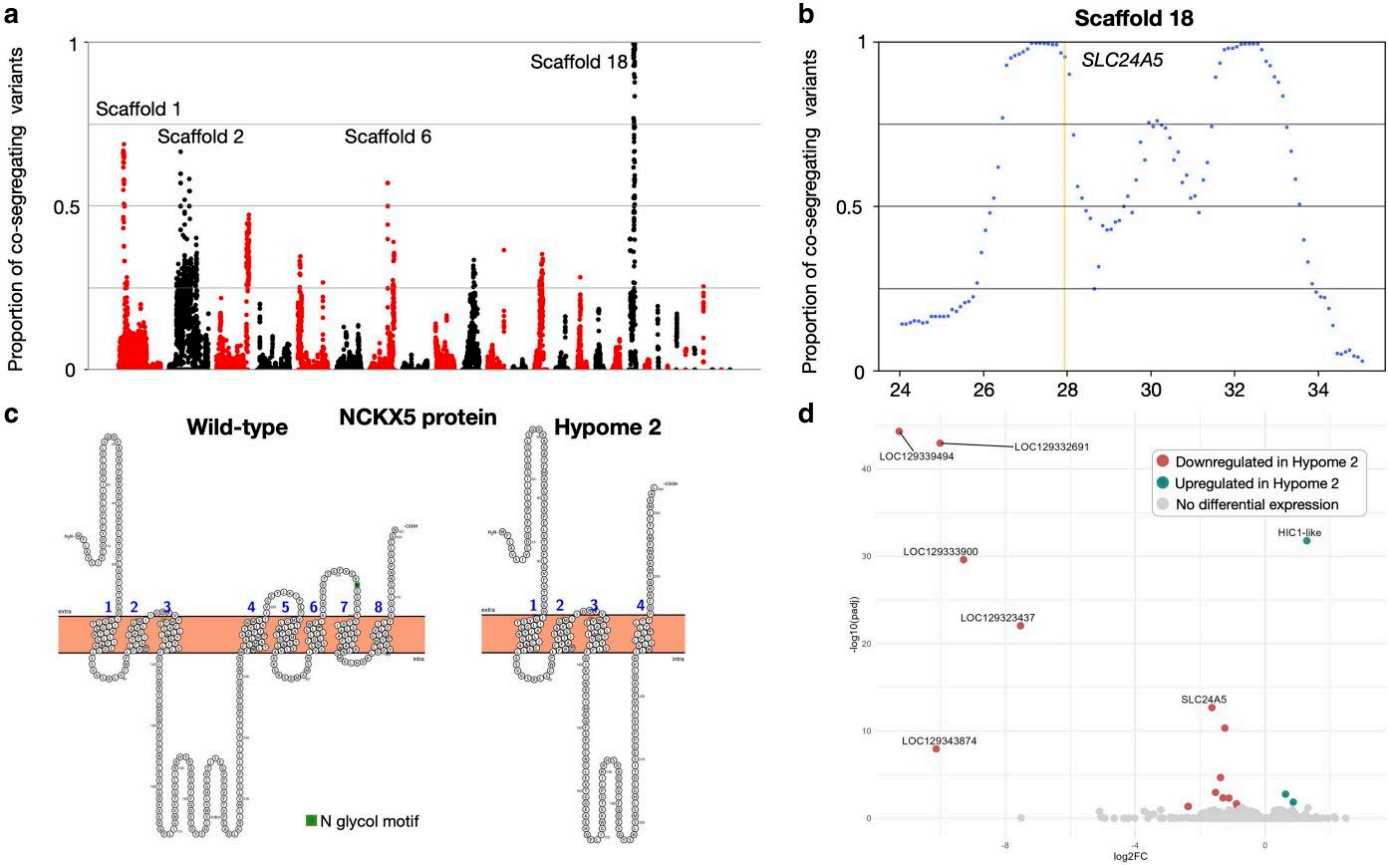
Genetic and transcriptomic analyses for the Hypome 2 leopard gecko morph. a) Proportion (*y*-axis) of quality-filtered biallelic SNP/MNP co-segregating with the Hypome 2 locus in the 4 genomic libraries compared to informative quality-filtered parental variants. Proportions are calculated for scaffolds >1 Mb, with a 1-Mb sliding window and a step of 100 Kb. Scaffolds (alternatively colored black and red) are ordered from longest to shortest. We label Scaffolds 1, 2, 6, and 18. b) Proportion of biallelic variants (SNP/MNP and indels) in 200-Kb intervals with a 50-Kb step co-segregating with the Hypome 2 locus on Scaffold 18. We highlight in yellow the position of *SLC24A5*. c) Schematic representation of transmembrane domains of *SLC24A5* from a wild-type and a Hypome 2 leopard gecko generated with PROTTER. d) Volcano plot depicting statistically significant gene expression changes between wild-type and homozygous Hypome 2 embryonic dorsal skin samples in terms of log2 fold-change (*x*-axis) and negative log10 of *P* value (*y*-axis). In cyan, the genes significantly upregulated in Hypome 2 and in red, the ones significantly downregulated (cutoff adjusted *P* value: 0.05).

Our mapping-by-sequencing analyses for the Hypome 2 phenotype revealed a high co-segregation interval (maximum of 99.6%) on Scaffold 18 and regions with lower levels (57% to 68%) on Scaffolds 1, 2, and 6 (Fig. 4a, Supplementary Table 2 in Supplementary File 2). The region on Scaffold 18 is split into 2 parts of high co-segregation: a 1.3 Mb region (from 26.7 to 28 Mb) and a 1.1 Mb region (31.7 to 32.8 Mb). The percentage of co-segregation in between ranges from 42% to 75% (Fig. 4b) and the number of co-segregating variants is lower (1,420/Mb) compared to the other 2 parts (2,584/Mb for a total of 6,201 co-segregating variants). We do not find long stretches of repetitive regions or of Ns in the in-between region that could account for the drop in the percentage of co-segregating variants.

The 2 regions of high co-segregation harbor 43 genes (Supplementary Table 11 in Supplementary File 2). Eight of thesegenes carry amino acid modifications that do not impact the predicted protein domains according to InterProScan (Supplementary File 3). A high-impact mutation is present in exon 6 (out of 8) of the *SLC24A5* gene resulting in a premature STOP codon through a C-to-T mutation changing a UGG codon to a UAG codon. The *SLC24A5* gene encodes NCKX5 (sodium/potassium/calcium exchanger 5). Based on the transmembrane (TM) domain prediction of PROTTER (Omasits et al. 2014), the Hypome 2 NCKX5 protein is missing 4 TM domains (Fig. 4c). *SLC24A5* is mostly expressed in pigment cells. It was first identified in zebrafish (Lamason et al. 2005) and since then has been found to be a major component in melanogenesis through the regulation of melanosome biogenesis (Wilson et al. 2013). A single missense mutation in humans is sufficient to disrupt the function of the protein (Bertolotti et al. 2016), so it is likely that the loss of 4 TM domains results in a severe disruption of the protein function in Hypome 2 leopard geckos, making it a strong candidate for this phenotype. In our single-cell data from embryonic skin, we observe that *SLC24A5* is strongly expressed in melanophores, and less so in the other chromatophores (Supplementary Fig. 6a in Supplementary File 1).

In parallel, we performed bulk RNA sequencing on dorsal skin samples from embryos at developmental stage 41 (Supplementary Table 4 in Supplementary File 2). Two breeding pairs produced 2 homozygous (*m^h2^*/*m^h2^*) and 6 wild-type (*m^h2^*/+ or +/+) embryos. In the PCA analyses, the samples do not cluster according to their phenotype (PC1: 68%—Supplementary Fig. 6b in Supplementary File 1). Eighteen genes are differentially expressed (Supplementary Table 12 in Supplementary File 2), and *SLC24A5* is the only one among them located in the genomic interval (Fig. 4d, Supplementary Fig. 6c in Supplementary File 1). The downregulation of *SLC24A5*, whose protein sequence is disrupted by the variant described above, could be caused by cellular feedback aimed at preventing the production of the defective protein, known as nonsense-mediated mRNA decay (Kurosaki et al. 2019). Of the 505 pigmentation-associated genes, none is differentially expressed in Hypome 2 embryonic skin, besides *SLC24A5*.

### *OCA2* is disrupted in Hypome 3 leopard geckos

**Fig. 5.**
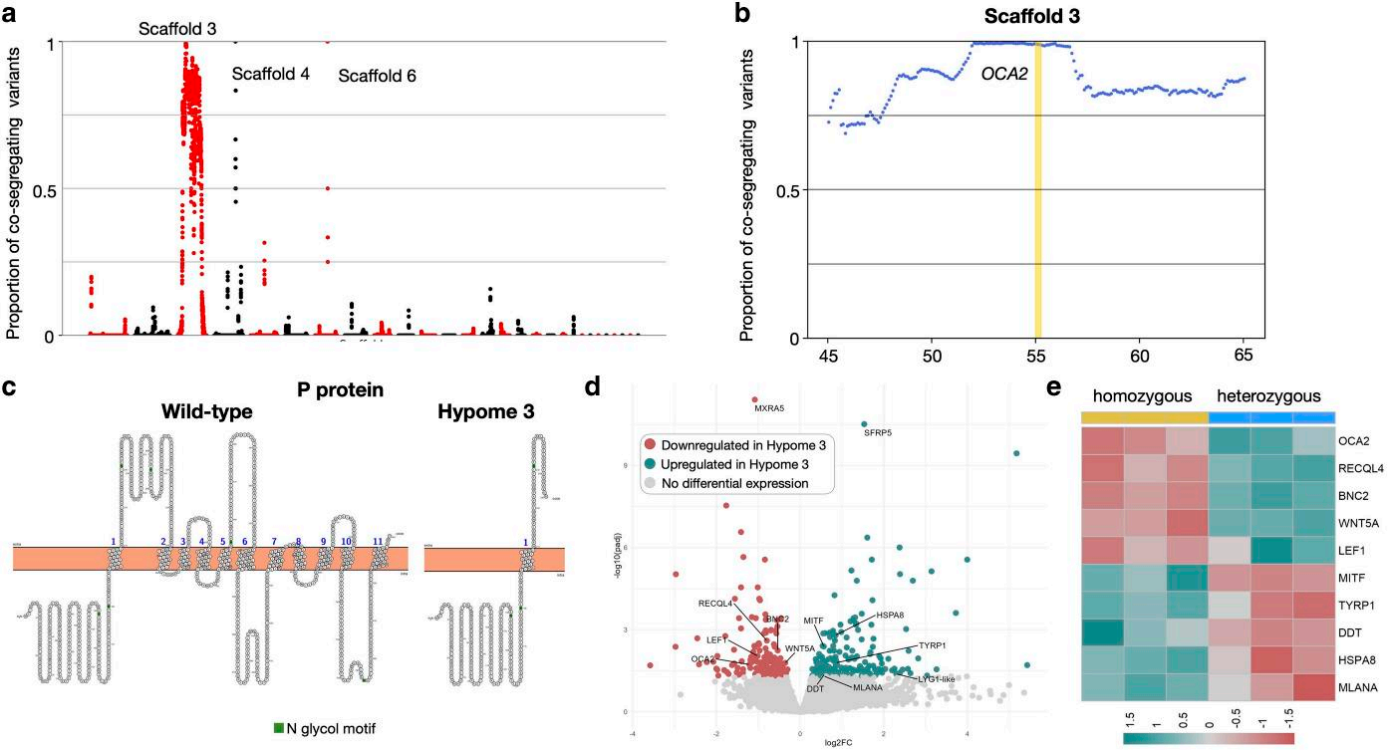
Genetic and transcriptomic analyses for the Hypome 3 leopard gecko morph. a) Proportion (*y*-axis) of quality-filtered biallelic SNP/MNP co-segregating with the Hypome 3 locus in the 4 genomic libraries compared to informative quality-filtered parental variants. Proportions are calculated for scaffolds >1 Mb, with a 1-Mb sliding window and a step of 100 Kb. Scaffolds (alternatively colored black and red) are ordered from longest to shortest. We label Scaffolds 3, 4, and 6. b) Proportion of biallelic variants (SNP/MNP and indels) in 200-Kb intervals with a 50-Kb step co-segregating with the Hypome 3 locus on Scaffold 3. We highlight in yellow the position of *OCA2*. c) Schematic representation of transmembrane domains of *OCA2* from a wild-type and a Hypome 3 leopard gecko generated with PROTTER. d) Volcano plot depicting statistically significant gene expression changes between heterozygous and homozygous Hypome 3 embryonic dorsal skin samples in terms of log2 fold-change (*x*-axis) and negative log10 of *P* value (*y*-axis). In cyan, the genes significantly upregulated in Hypome 3 and in red, the ones significantly downregulated (cutoff adjusted *P* value: 0.05). We label the differentially expressed genes associated with melanogenesis. e) Heatmap of genes that are associated with melanogenesis and differentially expressed in the Hypome 3 samples.

The mapping-by-sequencing analyses revealed a genomic interval of 4.8 Mb on Scaffold 3 (from 51.9 to 56.7 Mb) harboring the Hypome 3 causative mutation (Fig. 5, a and b, Supplementary Table 2 in Supplementary File 2). Two secondary regions with a high percentage of co-segregating variants are located on Scaffolds 4 and 6 but correspond to regions with a very small number of co-segregating variants (37 and 10, respectively). On the other hand, in the main interval there are 13,906 co-segregating variants (2,897/Mb). These variants result in amino acid substitutions in 12 out of the 46 genes present (Supplementary Table 13 in Supplementary Files 2 and 3), affecting the InterProScan protein domain prediction of CRACDL and AFF3. Both proteins lose a “Mobi-DB consensus disorder domain,” but the impact of these domain losses on protein function is hard to assess due to the lack of available literature about specific disordered regions and their functions. In addition, a high-impact variant is found within the *OCA2* gene. A G-to-A substitution changes a CGA codon to UGA in exon 6 (out of 23) introducing an early STOP codon. *OCA2* encodes the P protein whose exact function is unknown, although it is predicted to be a transmembrane transporter of tyrosine in melanocytes (Toyofuku et al. 2002). This protein is an essential factor of melanogenesis and has been linked to albinism in humans, zebrafish, and corn snakes (Beirl et al. 2014; Saenko et al. 2015). According to the TM domain prediction by PROTTER, 10 out of 11 TM domains are missing from the Hypome 3 P protein (Fig. 5c) very likely affecting its function. In our single-cell data from embryonic skin, we observed a restricted expression of *OCA2* in melanophores (Supplementary Fig. 7a in Supplementary File 1).

Bulk RNA sequencing was performed using dorsal skin samples from 3 homozygous (*m^h3^*/*m^h3^*) and 3 heterozygous (*m^h3^*/+) embryos at developmental stage 41 (Supplementary Table 4 in Supplementary File 2). The clustering by PCA roughly separates the samples by phenotype (Supplementary Fig. 7b in Supplementary File 1). Indeed, the heterozygous, rather than wild-type, samples used for the comparison might have an intermediate level of expression in-between that of wild-type and Hypome 3. 392 genes are nonetheless differentially expressed (Fig. 5d), 2 of which are within the genomic interval (Supplementary Table 14 in Supplementary File 2): *LOC129325423* (*LYG1*-like Lysozyme G1) and *OCA2*. The under-expression of *OCA2* could be due to the cellular feedback to the disrupted P protein, similarly to what we observe for Hypome 2 and *SLC24A5*. *LYG1* encodes a lysozyme protein that likely plays an antitumoral role via promotion of CD4+ T cells (Liu et al. 2017) but a link with skin pigmentation has not been established.

Out of the 505 pigmentation-associated genes, 9 are differentially expressed. Along with *OCA2*, *RECQL4* (RECQ Like Helicase 4), *BNC2* (Basonucline 2), *WNT5A* (Wnt Family Member 5A), and *LEF1* (Lymphoid Enhancer Binding Factor 1) are downregulated, whereas *MITF* (Microphthalmia-associated transcription factor)*, TYRP1* (Tyrosinase-related protein 1), *DDT* (D-Dopachrome Tautomerase), *HSPA8* (Heat Shock Protein Family A member 8), and *MLANA* (Melan-A) are upregulated in Hypome 3 embryonic skin (Fig. 5e, Supplementary Fig. 7c in Supplementary File 1). *BNC2* encodes a zinc finger protein associated with melanogenesis in humans (Jacobs et al. 2013) and plays an essential role for pigment cell development in zebrafish. It promotes melanophore and xanthophore development through the upregulation of *KITLGA*, *CSF1A,* and *CSF1B*, and of iridophores through a yet unknown process (Patterson and Parichy 2013). The ligand WNT5A prevents melanocyte proliferation in mammals (Zhang et al. 2013; Hong et al. 2025) and could participate in the Wnt signaling of pigment cells in the neural crest (Dorsky et al. 1998; Jin et al. 2001), although it has not been directly linked to this process. *RECQL4* is involved in iridophore development in the mimic poison frog (Rubio et al. 2024), whereas mutations in humans lead to the Rothmund–Thomson syndrome characterized by skin pigmentation defects, hinting at a potential role in melanogenesis (Lu et al. 2017). The transcription factor LEF1 regulates the expression of the melanin-producing enzyme TYR (Wang et al. 2015) and could also play a role in chromatophore differentiation in the neural crest via the activation of MITF (Dorsky et al. 2000). Among the upregulated genes, MITF is a transcription factor involved in chromatophore differentiation (Korzeniwsky et al. 2025) and more particularly in reptiles in the differentiation of melanophores and xanthophores (Ullate-Agote and Tzika 2021; Tzika 2024). It is also known to regulate the expression of melanogenesis enzymes, such as TYR, TYRP1, and DCT (Goding and Arnheiter 2019). TYRP1 and DDT participate in melanin synthesis (Odh et al. 1993; Cheli et al. 2010), and MLANA is involved in melanosome biogenesis and is essential for the proper function of tyrosinase through helping its folding, trafficking, and stability (De Maziere et al. 2002). The role of HSPA8 in pigmentation has not been fully uncovered, but it has been linked to color adaptation at different temperatures (Kulkeaw et al. 2011). Overall, the associated differential expression of these genes suggests that: (i) the differentiation of melanophores is impacted possibly resulting in a decrease of their number and (ii) the expression of melanogenesis factors is increased in the melanophores still present.

### Melanosomes are abnormal in hypomelanistic squamate morphs

**Fig. 6.**
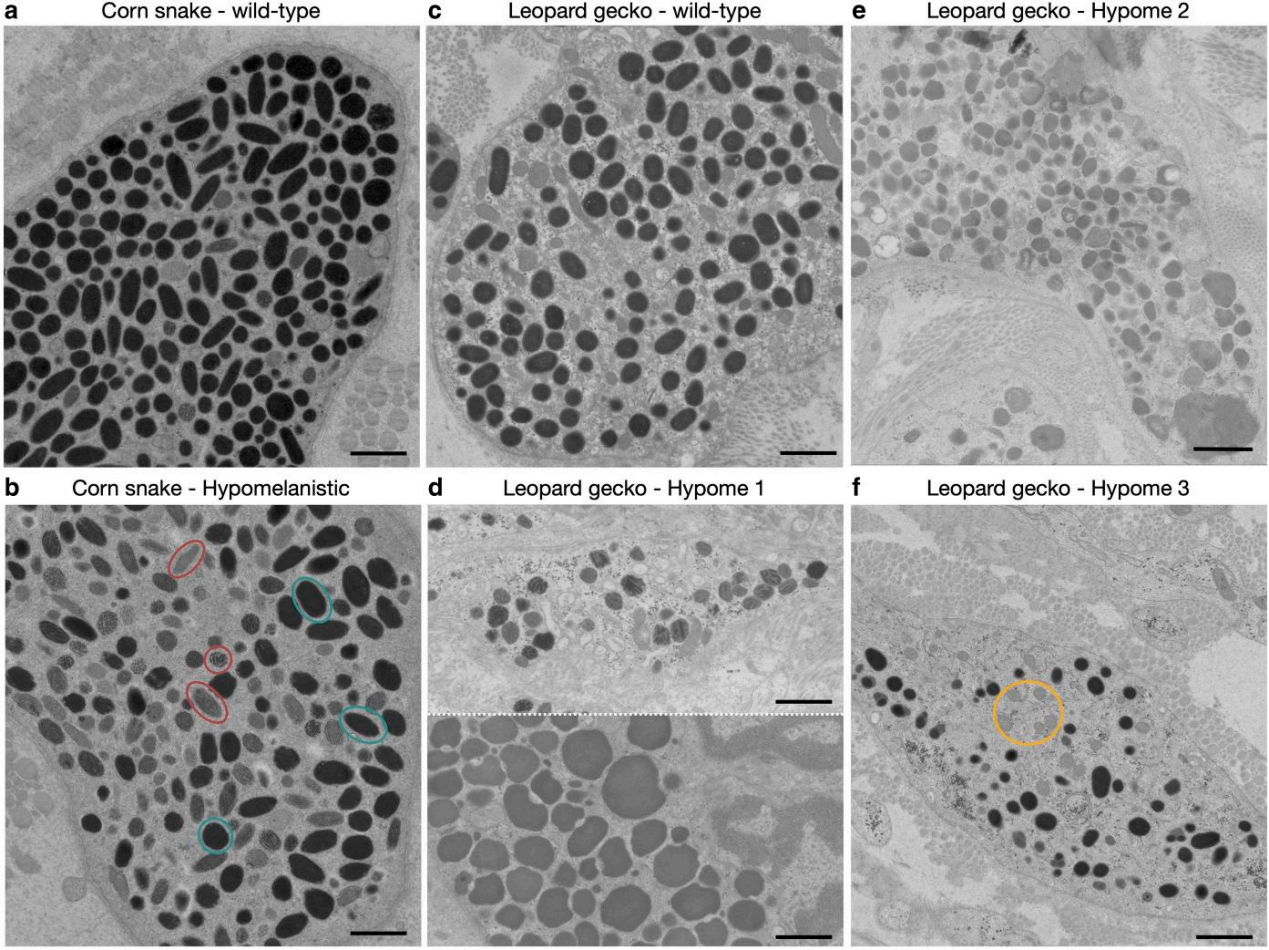
Abnormal melanogenesis in 4 hypomelanistic morphs. Transmission electron microscopy images of melanophores from wild-type (a) and hypomelanistic (b) corn snakes and from wild-type (c), Hypome 1 (d), Hypome 2 (e), and Hypome 3 (f) leopard geckos. In Hypome 1 leopard gecko (d), we observe both small melanosomes at an early stage of maturation (top panel) and enlarged mature ones (bottom panel). Cyan and red circles in (a) highlight mature and early-stage melanosomes, respectively. The orange circle in (f) encloses endosomes that are likely modified melanosomes. Scale bars: 1 μm.

To elucidate the impact of each mutation on the melanophores of the 4 hypomelanistic morphs, we prepared semi-thin sections from the dorsal skin of adults for transmission electron microscopy (Fig. 6). In the Hypomelanistic corn snake, the melanosomes have a similar shape and size as those found in the wild-type but most of them appear at an early stage of maturation (Fig. 6, a and b). Indeed, in a single section of a hypomelanistic melanophore, we observe 48% of early-stage melanosomes, compared to 2.4% for the wild-type. Although the area imaged with electron microscopy is very small to make conclusions for the entire tissue, we noted that melanophores were sparse in the samples observed. In Hypome 1 leopard geckos (Fig. 6d), we found both enlarged and reduced melanosomes, compared to the wild-type (Fig. 6c), with most of the small melanosomes being at an early stage of maturation. We hypothesize that a regulatory mutation in *TYR* is responsible for the hypomelanistic phenotype in corn snakes and we identified a missense mutation in the same gene as responsible for the Hypome 1 phenotype in leopard geckos. If our hypothesis is correct, the differences in melanosome structure between the 2 morphs could be due to the type of mutations, as well as the differential expression of other pigmentation-associated genes in each species.

In Hypome 2 leopard geckos, the shape and size of melanosomes is heavily impacted, but the maturation process is not affected as the melanosomes appear fully melanized (Fig. 6e). For this morph, we identified a disruptive mutation in *SLC24A5*, which codes for NCKX5, a potassium-dependent sodium-calcium exchanger potentially situated on the mitochondrial membrane. It is thought to regulate melanosomal Ca^2+^ homeostasis and subsequently the formation of PMEL fibrils and the maturation of the melanosomes (Zhang et al. 2019). In cultured melanocytes, knocking down *SLC24A5* disrupts melanogenesis but this does not seem to be the case in Hypome 2, where melanin is produced and the melanosome morphology is primarily impacted. In Hypome 3 leopard geckos, the melanosome shape and size are abnormal, and their number seems to be reduced within the observed melanophores (Fig. 6f). Based on the amelanistic phenotype of other vertebrate *OCA2* mutants, one would expect to primarily find early-stage melanosomes, but this is not the case in Hypome 3. Note that in amelanistic corn snakes that also carry a disruptive *OCA2* mutation, melanophores are difficult to identify in the absence of mature melanosomes and only a few cells contain potentially early-stage melanosomes (Supplementary Fig. 8 in Supplementary File 1).

## Discussion

Hypomelanism and amelanism have been extensively studied in mammals, in particular the impact of mutations on human and mouse skin pigmentation and the associated diseases and syndromes. Comprehensive studies on other vertebrate lineages highlight mostly the involvement of the same key factors but lack precision on the exact role of these elements in each lineage and how their disruption or dysregulation affects the melanophore differentiation and maturation. In this context, we undertook the genetic characterization of 4 hypomelanistic squamate morphs, 1 for the corn snake and 3 for the leopard gecko. We identified candidate mutations in 3 genes with known function in pigmentation: (i) *TYR* encoding tyrosinase, an essential enzyme for melanin synthesis, for Hypomelanistic corn snakes and Hypome 1 leopard geckos, (ii) *SLC24A5* encoding NCKX5, an ion exchanger that regulates the PMEL fibril formation for Hypome 2, and (iii) *OCA2* encoding the P protein, a transmembrane transporter for tyrosine for Hypome 3 (Table 2). With our transcriptomic and imaging analyses, we aimed at elucidating the impact of these mutations. In addition to the proposed mutations for the 3 hypomelanistic leopard gecko morphs affecting the protein sequence, cis-regulatory may also contribute to the observed phenotypes. Functional analyses are needed to establish whether the proposed mutations are causative.

**Table 2.** Summary of the morphs, the candidate mutations, and the differentially expressed pigmentation-related genes.

	Hypomelanistic corn snake	Hypome 1 leopard gecko	Hypome 2 leopard gecko	Hypome 3 leopard gecko
Pet trade name	Hypomelanistic	Tremper albino	Rainwater albino	Bell albino
Alleles	*m^h^*	*m^h1^*	*m^h2^*	*m^h3^*
Candidate gene	regulatory mutation *TYR*↓	missense mutation *TYR*	disruptive mutation *SLC24A5*↓	disruptive mutation *OCA2*↓
Differentially expressed pigmentation-related genes	*BACE2*↓, *SYTL2*↓, *DDT-like*↑	*MYO7A*↑, *AP1S3*↑, *CTSD*↑, *APOE*↓, *SULF1*↓, *LEF1*↓, *EDNRB*↓, *XDH*↑	none	*RECQL4*↓, *BNC2*↓, *WNT5A*↓, *LEF1*↓, *MITF*↑, *TYRP1*↑, *DDT*↑, *HSPA8*↑, *MLANA*↑

The arrows next to the gene names indicate up- and downregulated genes in the homozygous mutants.

Using transmission electron microscopy, we investigated the impact of the mutations on the subcellular morphology of melanosomes. As a result of the downregulation of *TYR* in hypomelanistic snakes, the maturation of melanosomes is impacted, with early-stage melanosomes present in adult tissue. By compiling a melanogenesis-related set of 505 genes, we could interrogate our transcriptomic data and identify 3 additional dysregulated genes in the hypomelanistic embryonic skin that primarily play a role in melanin synthesis and proper melanosome biogenesis. For the Hypome 1 leopard gecko, we propose that a missense mutation in *TYR* is associated with the phenotype. In the subcellular level, not only the maturation but also the shape and size of the melanosomes is impacted. The mutated *TYR* might not be solely responsible for this abnormal phenotype. Indeed, our transcriptomic analyses reveal that additional pigmentation-related genes are dysregulated, affecting melanin production, melanosome motility, and melanophore migration. This implies that the number of melanophores could also be impacted. In Hypome 2, the disruption of *SLC24A5* primarily impacts the shape of the melanosomes. As no other pigmentation-related gene appears dysregulated, we hypothesize that the main function of NCKX5 in leopard geckos is linked to proper melanosome formation. In Hypome 3, the *OCA2* disruption translates to a truncated P protein and several genes involved in melanin synthesis and melanophore differentiation are differentially expressed. This could explain the presence of premature melanosomes in the adult tissue that maintain their proper shape and implies a reduced number of melanophores. The subcellular phenotype matches observations in zebrafish *OCA2* mutants (Beirl et al. 2014), where the number of melanophores decreases as also suggested by our transcriptomic analyses. In contrast, corn snake *OCA2* mutants are amelanistic and their melanophores cannot be identified with confidence in the absence of mature melanosomes.

Gamble et al. (2006) showed that tyrosinase is active in the adult skin of the 3 hypomelanistic leopard gecko morphs discussed here by performing a DOPA test. This observation agrees with our findings. In Hypome 1, the single amino acid substitution is more likely to alter the enzyme's efficiency than to disrupt its function. In the other 2 morphs, the tyrosinase is intact. Brown et al. (Brown et al. 2022) suggested that missense mutations in *TYR* and disruption of *OCA2* are responsible for 2 albino phenotypes in ball pythons. The *TYR* missense mutations in Albino ball pythons are near the mutated site of Hypome 1 leopard geckos, providing further support to our findings. Ball python *TYR* mutants have a paler pigmentation than the *OCA2* mutants. This is the case for Hypome 1 when compared to Hypome 3, the 2 leopard gecko morphs with *TYR* and *OCA2* mutations, respectively. Our study relies on developmental stages for the transcriptomic analyses and the adult stage for the imaging. We previously described the dynamic behavior of chromatophores in the leopard gecko skin as the animals mature from hatchlings with transverse bands to adults with a spotted pattern (Ullate-Agote and Tzika 2024). A systematic analysis of this ontogenetic change is necessary to understand how each mutation potentially impacts the mobility of melanophores during this dynamic phase.

Our findings demonstrate that similar mutations can result in different skin pigmentation phenotypes in different species, highlighting the modularity of the melanogenesis process. Through the integration of genomic, transcriptomic, and imaging analyses, we emphasize the importance of investigating how macroscopic traits, such as overall skin pigmentation, relate to the subcellular structure of chromatophores. Furthermore, associated changes in the expression levels of other pigmentation-related genes may play a crucial role in shaping the final phenotype. Causative mutations are often regarded as solely responsible for the phenotypic alterations observed, yet these mutations might cause a more generalized misregulation of the chromatophore biology. When comparing different morphs, commonalities can be detected. For example, *LEF1*, an enhancer of *TYR* activity and chromatophore differentiation, is downregulated in both Hypome 1 and Hypome 3. Thus, disrupted melanogenesis might impact chromatophore differentiation as well. On the other hand, we can speculate that the upregulation of *DDT* in Hypomelanistic corn snakes and Hypome 3 leopard geckos could compensate for the defects in melanin production. Overall, these multifactorial interactions can enhance our understanding of the functions and synergies among the different pigmentation elements that collectively produce skin colors and patterns.

## Supplementary Material

iyaf236_Supplementary_Data

## Data Availability

The datasets generated and analyzed in the current study are available in NCBI: Hypomelanistic corn snake genomic sequencing PRJNA1224566 and RNA-Seq GSE289621; Hypome1, Hypome2, and Hypome3 leopard gecko genomic sequencing PRJNA1225496 and RNA-Seq GSE289890. The LiftOn annotation of the leopard gecko genome (NCBI accession GCA_050574515.1) is available on Yareta: https://doi.org/10.26037/yareta:p7pj6gc3w5bepjq7ysjyxd3piy. Supplemental material available at GENETICS online. Supplementary figures are available in Supplementary File 1, and the Supplementary tables are in Supplementary File 2. Protein sequence alignments are provided in Supplementary File 3.
