# Supplementary material for: Candidate genes underlying hypomelanistic morphs in squamate reptiles
Source: Genetics. 2025 Nov 3;232(1):iyaf236. doi: 10.1093/genetics/iyaf236 (PMC12774848; doi:10.1093/genetics/iyaf236)
Supplement: iyaf236_Supplementary_Data [file iyaf236_supplementary_data.zip › Supplementary_File_1_GENETICS-2025-308529.pdf]

Candidate genes underlying hypomelanistic morphs in squamate reptiles

Pierre Beaudier [1], Asier Ullate-Agote [1,2], Athanasia C. Tzika[1*]

[1] Laboratory of Artificial and Natural Evolution, Department of Genetics & Evolution, University of Geneva, Geneva 1205, Switzerland

[2] current address: Biomedical Engineering Program, Center for Applied Medical Research (CIMA), Universidad de Navarra, Instituto de Investigación Sanitaria de Navarra (IdiSNA), Pamplona 31009, Spain

[*] Corresponding author

Email: athanasia.tzika@unige.ch (ACT)

Supporting information

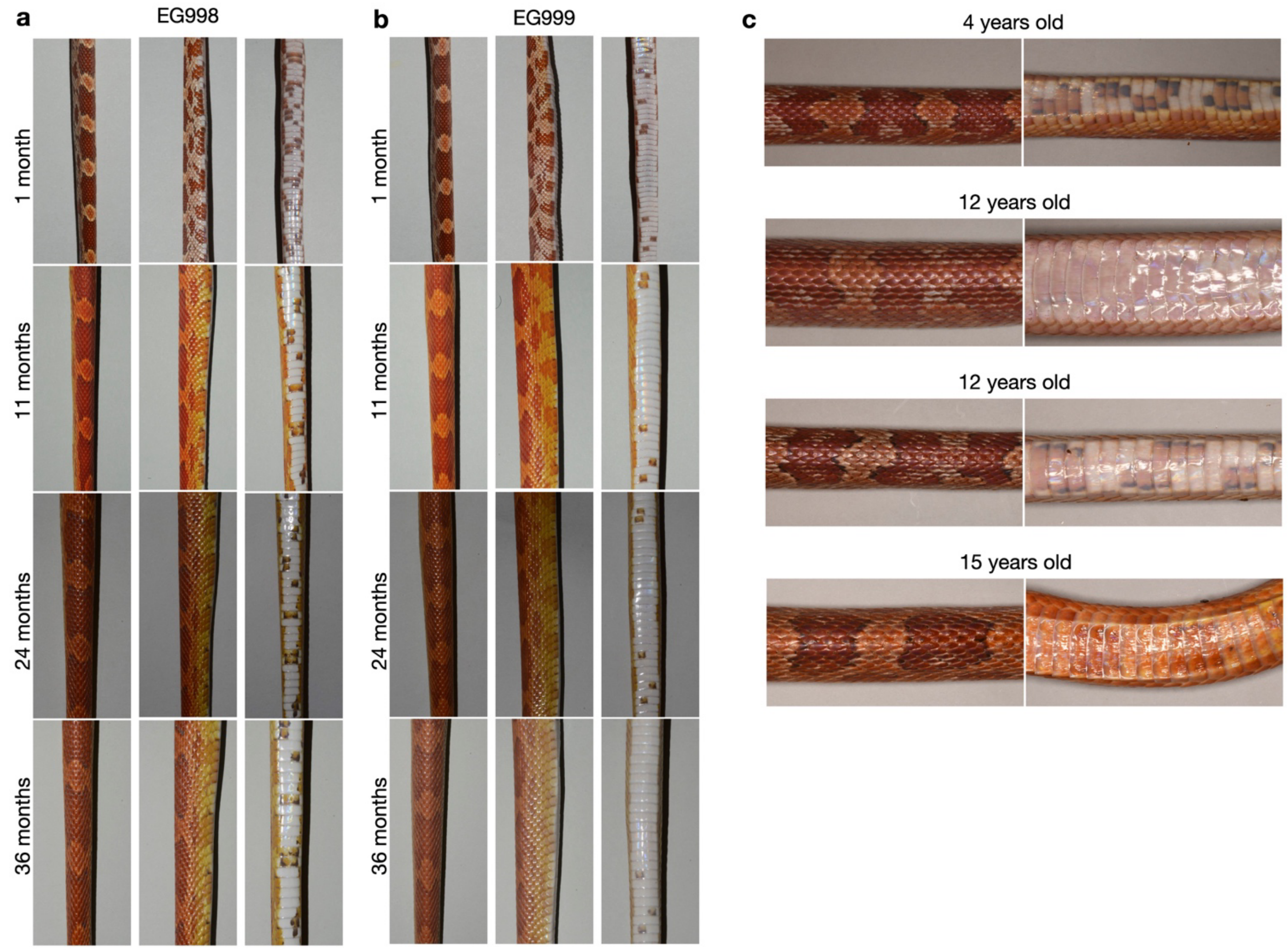

Supplementary Figure 1. Imaging of Hypomelanistic corn snakes. a,b) Photos of the dorsal, lateral, and ventral side from the posterior part of the body (near the cloaca) of two Hypomelanistic individuals at four time points post hatching. **c)** Colour variation in four Hypomelanistic corn snakes of four to fifteen years old.

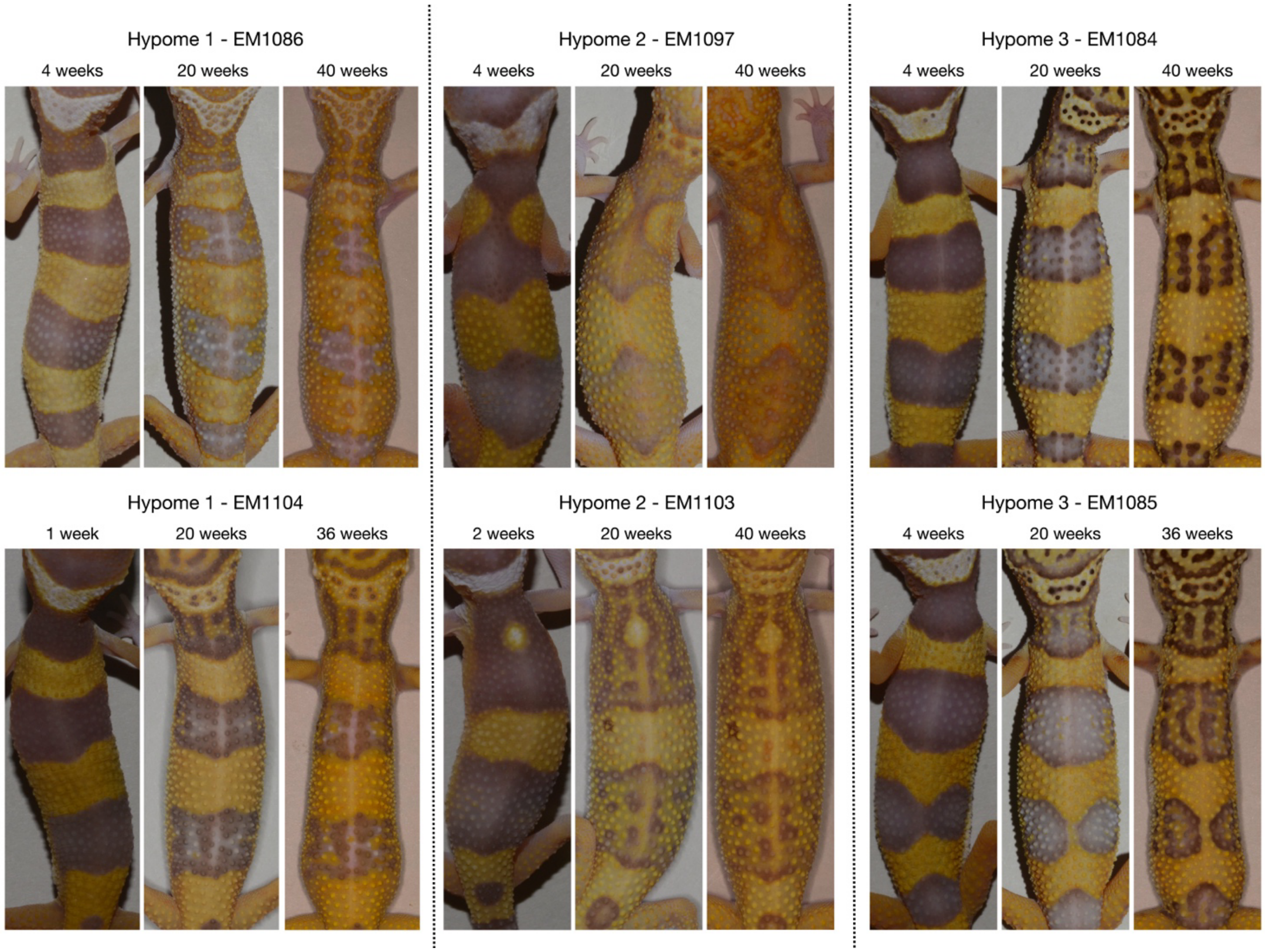

Supplementary Figure 2. Imaging of hypomelanistic leopard geckos. Dorsal side photos of the dorsal side of two Hypome 1, two Hypome 2 and two Hypome 3 leopard geckos at three time points post hatching.

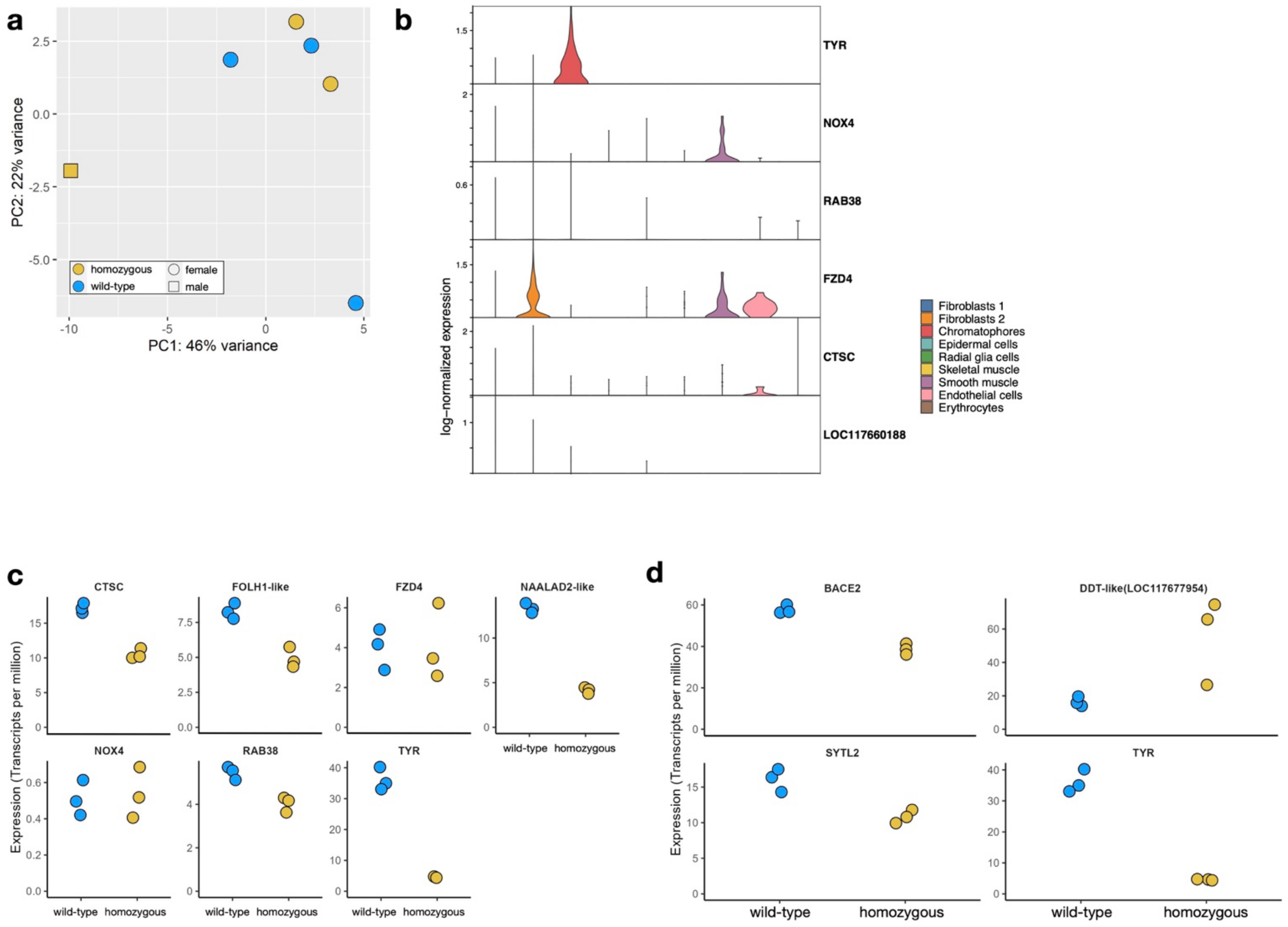

Supplementary Figure 3. Bulk RNA-seq differential expression analyses for Hypomelanistic corn snakes. a) Principal component analysis of the six RNA-seq samples. **b)** Violin plot of the log-normalised expression levels of genes in the Hypomelanistic interval and differentially expressed in Hypomelanistic samples in dorsal skin cells from an embryo at embryonic day E25 when no melanin is visible. **c)** Transcripts per million detected for four genes located in the Hypomelanistic corn snake interval. **d)** Transcripts per million detected for four melanogenesis-related genes in wild-type and homozygous samples.

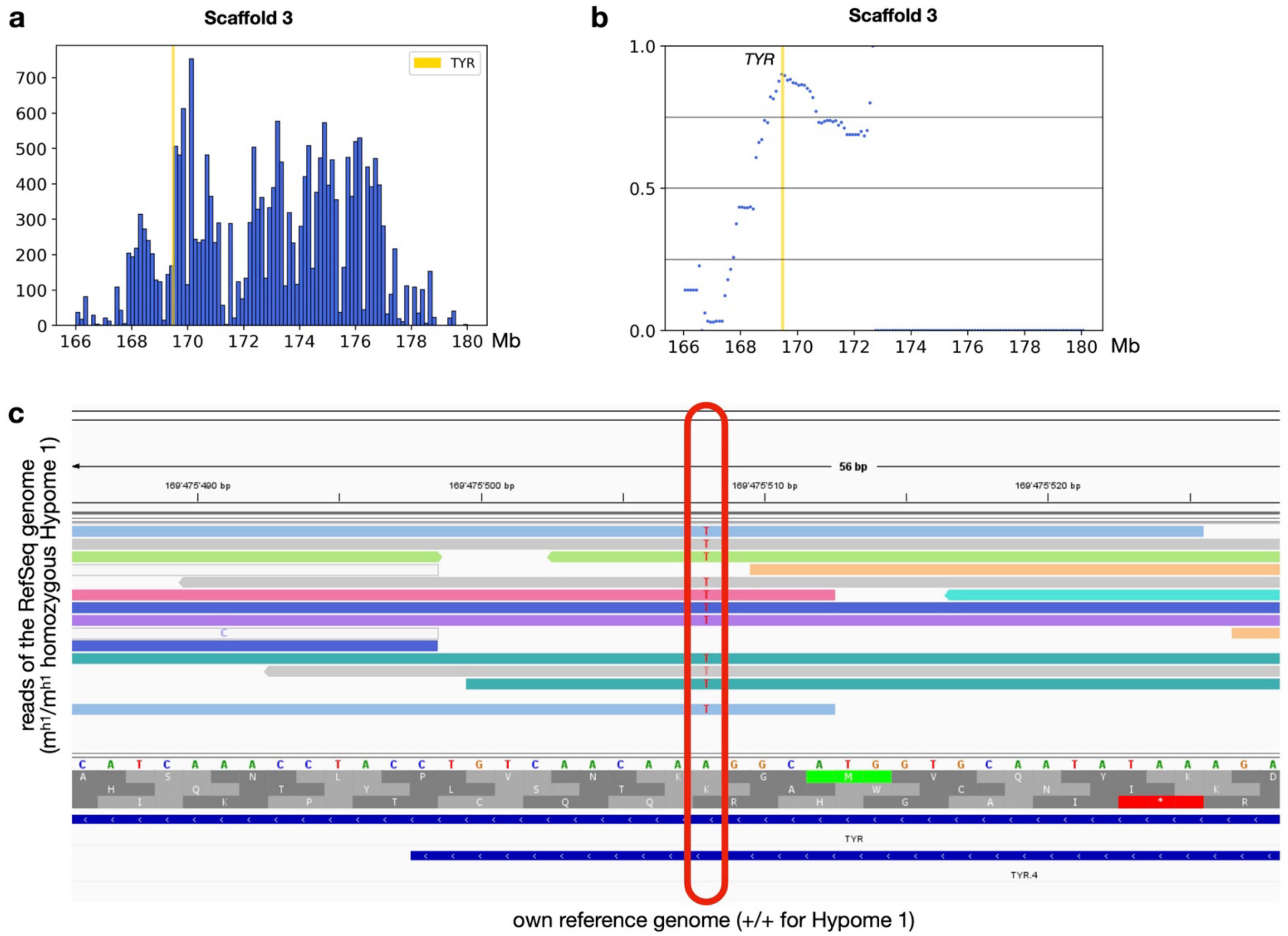

Supplementary Figure 4. Genetic analyses for the Hypome 1 leopard gecko morph. a) Number of biallelic variants (SNP/MNP and indels) in 500-kb intervals cosegregating with the Hypome 1 locus in the genomic interval. **b)** Proportion of biallelic variants (SNP/MNP and indels) in 200-Kb intervals with a 50-Kb step co-segregating with the Hypome 1 locus on Scaffold 3. Sequencing reads used for the assembly of the RefSeq genome were included in the analyses because they originated from a homozygous Hypome 1 individual) We highlight in yellow the position of *TYR*. **c)** Alignment of the RefSeq reads to our own assembly from a wild-type individual. We highlight the single-nucleotide polymorphism in the *TYR* coding sequence.

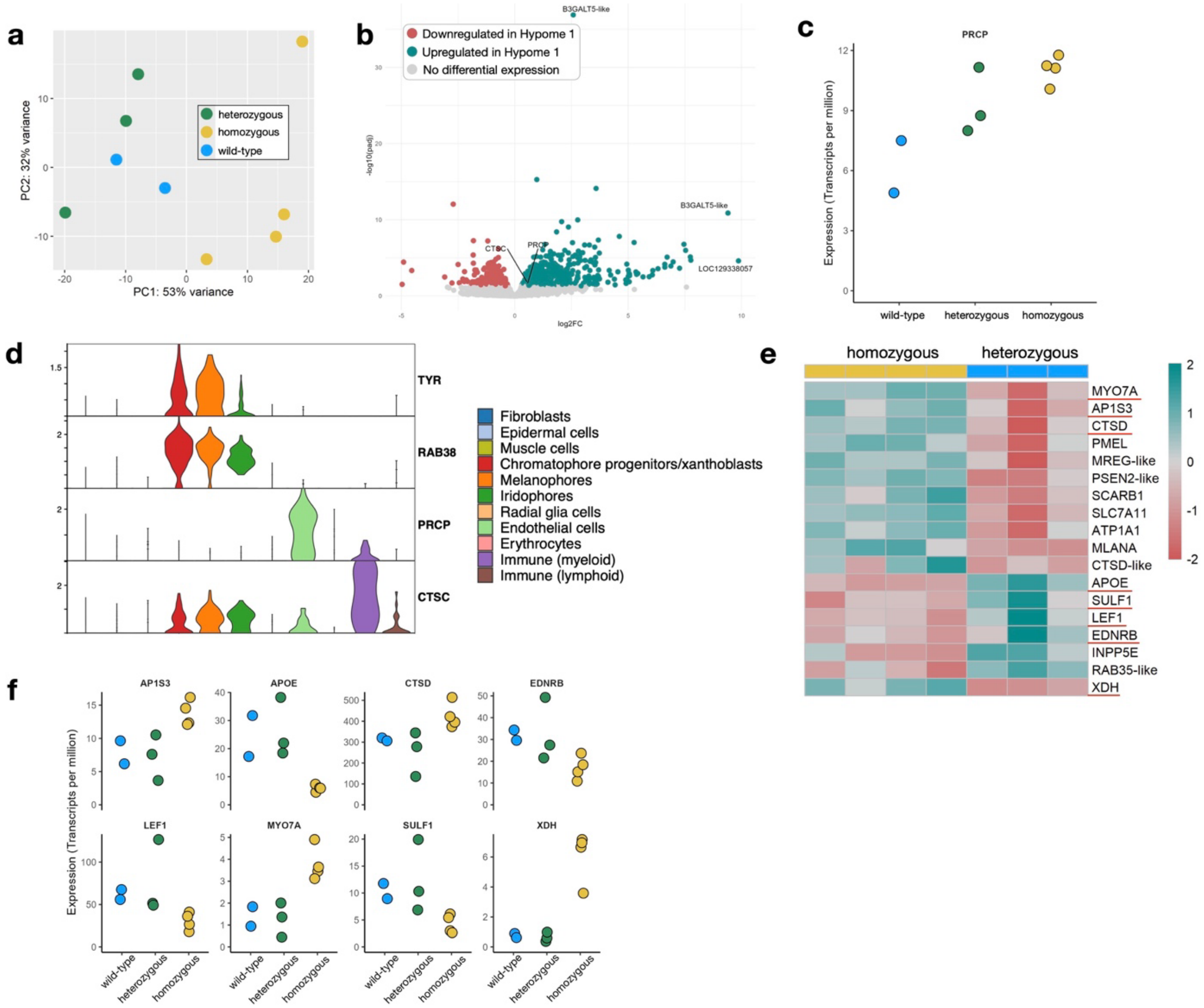

Supplementary Figure 5. Transcriptomic analyses for the Hypome 1 leopard gecko morph. a) Principal component analysis of the nine RNA-seq samples. **b)** Volcano plot depicting statistically significant gene expression changes between heterozygous and homozygous Hypome 1 embryonic dorsal skin samples in terms of log2 fold-change (x-axis) and negative log10 of *p* value (y-axis). In cyan, the genes significantly upregulated in Hypome 1 and in red, the ones significantly downregulated (cut-off adjusted *p* value: 0.05). We label the two genes located in the genomic interval. **c)** Transcripts per million detected for *PRCP*. **d)** Violin plot of the log-normalised expression levels of *TYR, RAB38, PRCP,* and *CTSC* in dorsal skin cells from an embryo at developmental stage 40 when melanin production initiates. **e)** Heatmap of differentially expressed genes in Hypome 1 samples (versus heterozygous) which are associated with melanogenesis. Underlined in red the common genes with the homozygous vs wild-type comparison. Note that *CTSD* and *CTSD-like* are located on different chromosomes and their protein sequence identity is 51%. **f)** Transcripts per million detected for eight melanogenesis-related genes in wild-type, heterozygous and homozygous Hypome 1 samples.

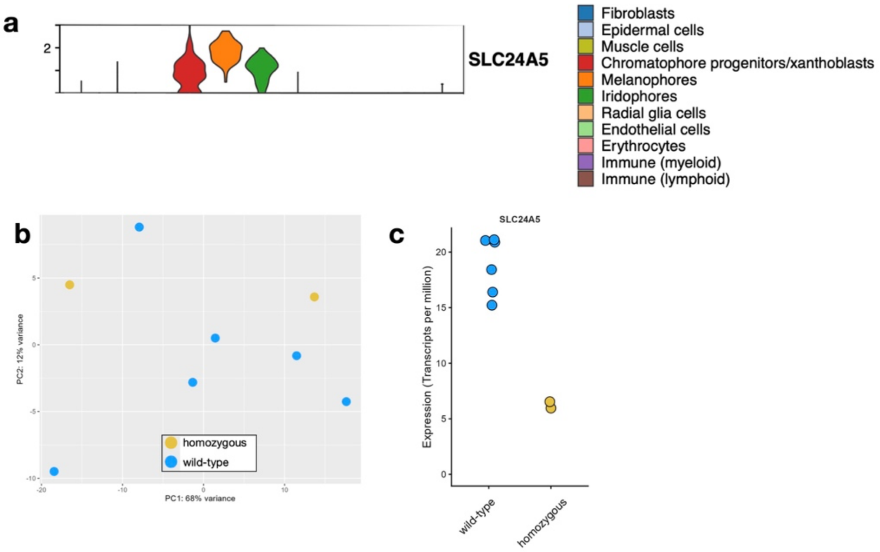

Supplementary Figure 6. Bulk RNA-seq differential expression analyses for Hypome 2 leopard geckos. a) Violin plot of the log-normalized expression levels of *SLC24A5* in dorsal skin cells from an embryo at developmental stage 40 when melanin production initiates. **b)** Principal component analysis of the eight RNA-seq samples. **c)** Transcripts per million detected for *SLC24A5* in wild-type and homozygous samples.

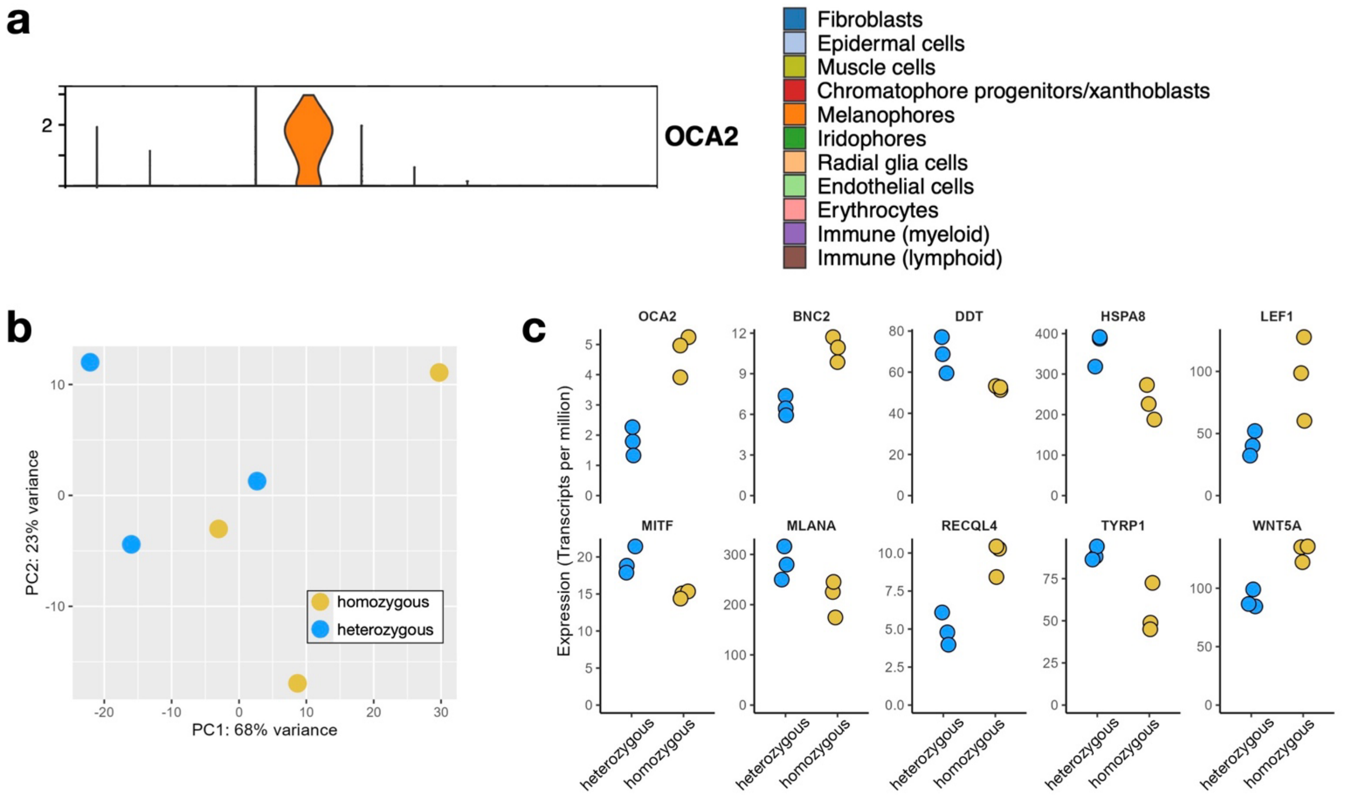

Supplementary Figure 7. Bulk RNA-seq differential expression analyses for Hypome 3 leopard geckos. a) Violin plot of the log-normalised expression levels of *OCA2* in dorsal skin cells from an embryo at developmental stage 40 when melanin production initiates. **b)** Principal component analysis of the six RNA-seq samples. **c)** Transcripts per million detected for ten melanogenesis-related genes in heterozygous and homozygous samples.

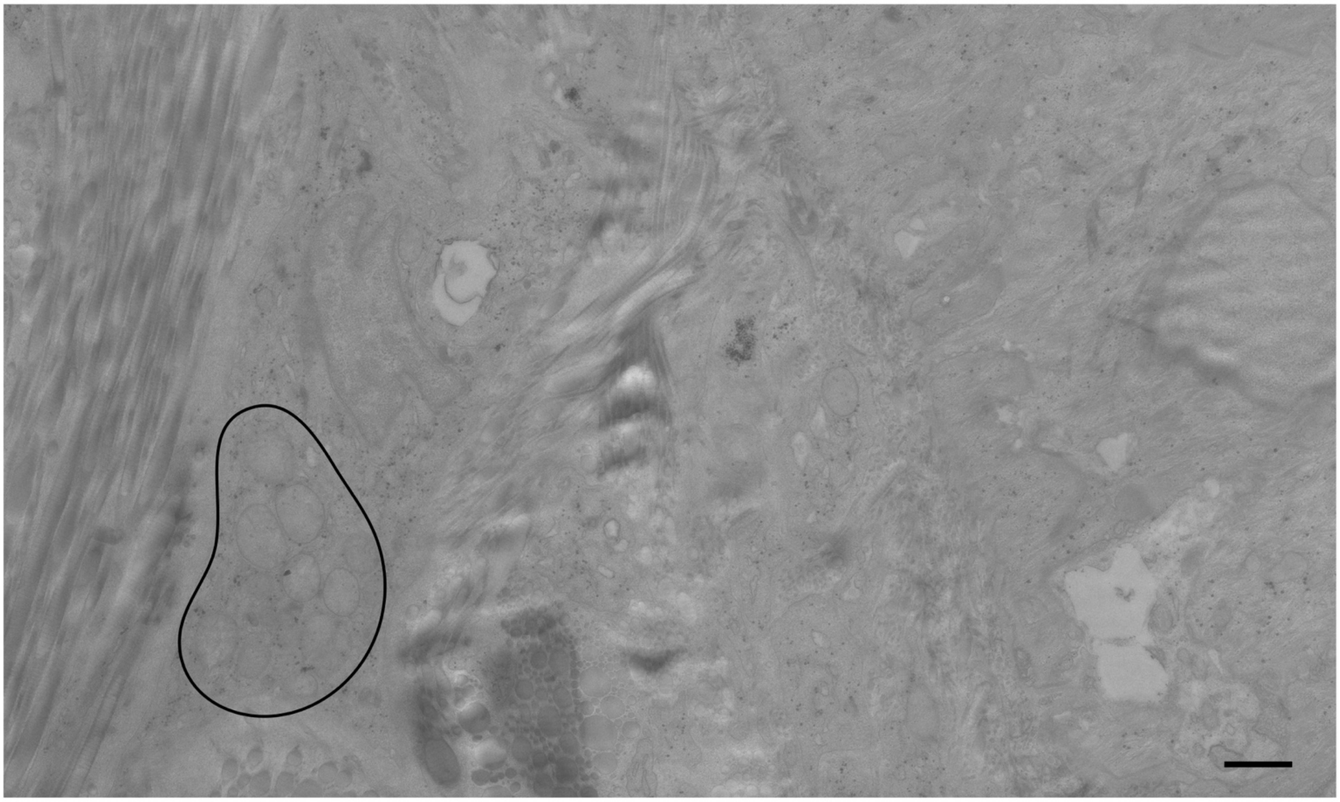

Supplementary Figure 8. Semi-thin section of dorsal skin from an Amelanistic corn snake. In a few dermal cells, we observe vesicles that appear empty (no striations, no melanin accumulated) that could correspond to melanosomes (black circle). No cells with early-stage or mature melanosomes could be seen. Scale bar: 1 µm.
